# Supplementary material for: Potential sublimating exocomets around the young star PDS 70
Source: Nat Commun. 2026 Aug 24;17:8894. doi: 10.1038/s41467-026-76880-y (PMC13503679; doi:10.1038/s41467-026-76880-y)
Supplement: Supplementary file 1 [file 41467_2026_76880_MOESM1_ESM.pdf]

Potential sublimating exocomets around the young star PDS 70

Aline Novais[1*], H. Jens Hoeijmakers[1], Alexandra Stockwell Murphy[1], Bibiana Prinoth[1,2], Anders Johansen[1,3], Klaudia Jaworska[1], Luan Ghezzi[4]

[1]Division of Astrophysics, Department of Physics, Lund University, Box 118, 221 00, Lund, Sweden.
[2]European Southern Observatory, Karl-Schwarzschild-Strasse 2, 85748, Garching, Germany.
[3]Center for Star and Planet Formation, Globe Institute, University of Copenhagen, Øster Voldgade 5–7, 1350, Copenhagen, Denmark.
[4]Valongo Observatory, Federal University of Rio de Janeiro, Ladeira do Pedro Antonio, 43, 20080-090, Rio de Janeiro, Brazil.

*Corresponding author(s). E-mail(s): aline.novais@fysik.lu.se;

Supplementary Information

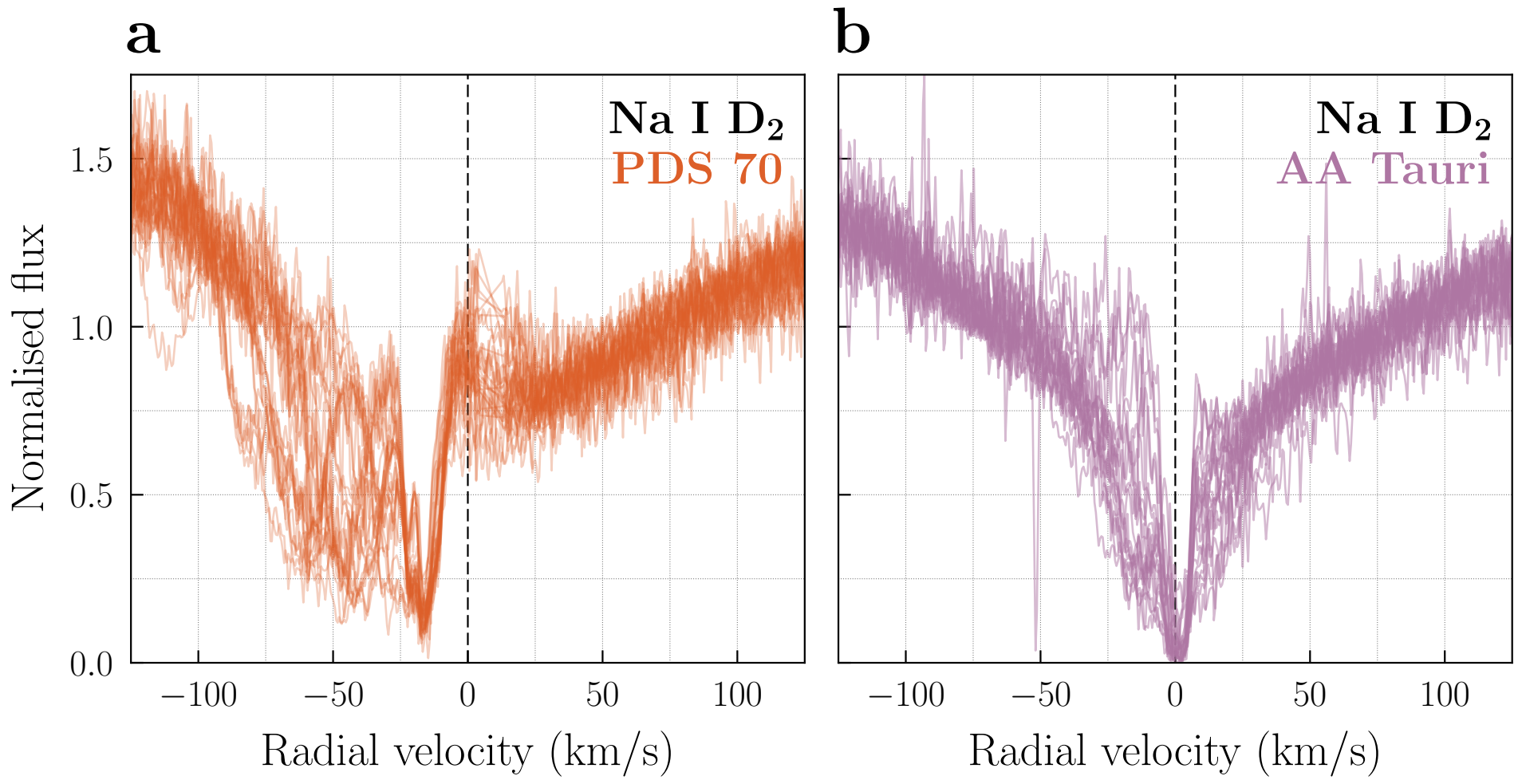

Supplementary Figure 1: **Sodium lines in PDS 70 and AA Tauri.** Radial velocities derived from HARPS data around the Na I D_2 line (588.996 nm) of K7 T Tauri stars PDS 70 (**a,** orange) and AA Tauri (**b,** purple). Spectra are centred (dashed line) at a system radial velocity of 6.01 km/s for PDS 70 (see Methods, subsection Derivation of the systemic velocity) and 17.20 km/s for AA Tau [1]. Data of PDS 70 consist on 32 spectra taken on 12 nights between 2018-03-29 and 2018-05-13 (see Table 1 in the main text for Programme IDs). Data of AA Tau consist on 21 spectra taken between 2004-10-10 and 2004-12-02 (Programme ID 074.C-0221, PI Bouvier). Source data are provided (see Data Availability in the main text).

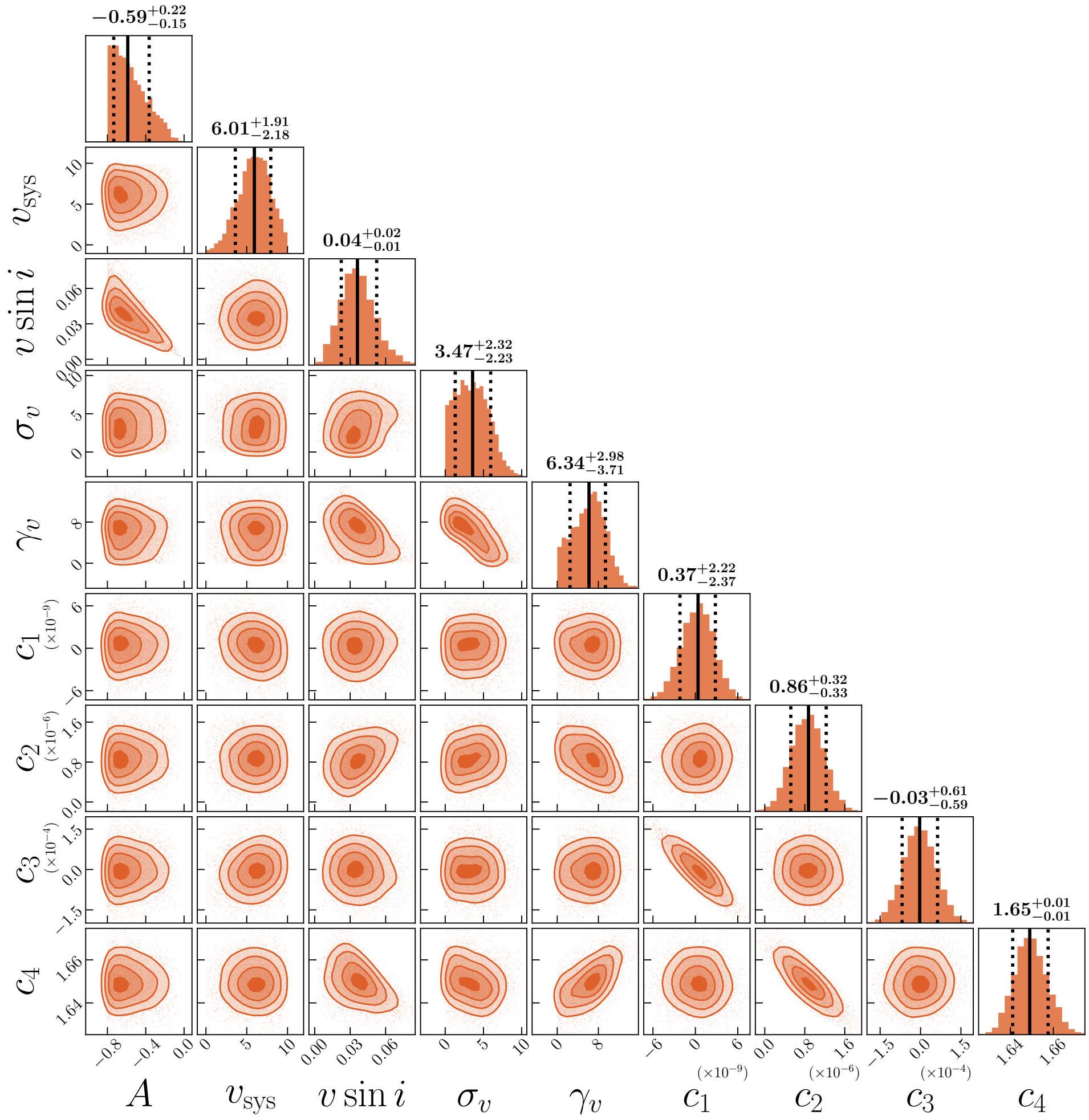

Supplementary Figure 2: **Posterior of the PDS 70 systemic velocity.** Posterior distributions of the cross-correlated 2018 HARPS spectra with a PHOENIX model spectrum to calculate the PDS 70 systemic velocity. A is the amplitude of the Voigt line, $v_{\rm sys}$ is the systemic velocity in km/s, $v \sin i$ is the projected rotational velocity of the star in km/s (where i is the inclination of the stellar rotation axis), σ_v is the Gaussian line width in km/s, γ_v is the Lorentzian width in km/s, and c_1, c_2, c_3, and c_4 are the polynomial coefficients (see Methods, subsection Derivation of the systemic velocity). Source data are provided (see Data Availability in the main text).

Supplementary Table 1: Priors used to fit the Na I line components i, according to the line (first column), and the continuum. μ is the centre wavelength of the line in nm, σ_v is the Gaussian line width in km/s, γ_v is the Lorentzian line width in km/s, τ is the optical depth, and f is the surface fill fraction. Note that the photospheric absorption line and the stationary emission line have the surface fill fraction fixed to 1. c_0 and c_1 are the offset and linear polynomial coefficients, respectively, for the first spectral section (around Na I D_2), while d_0 and d_1 are the same coefficients for the second section (around Na I D_1). All priors are assumed to be uniform across the range of values, with the exception of the optical depth, which uses log-uniform priors. This table is also provided in electronic form (see source data in Data Availability in the main text).

Line component	μ	σ_v	γ_v	τ	f
stellar photospheric absorption	[589.156, 589.160]	[1, 20]	[20, 60]	[0.001, 5]	1
stationary emission	[589.156, 589.160]	[1, 40]	-	[0.001, 5]	1
stationary absorption 1	[589.113, 589.117]	[0.001, 3]	-	[0.001, 5]	[0, 1]
stationary absorption 2	[589.125, 589.129]	[0.001, 3]	-	[0.001, 5]	[0, 1]
variable absorption	-	-	-	[0.001, 5]	[0, 1]

Continuum	c_0	c_1	d_0	d_1	-
polynomial coefficient	[1, 2]	[−0.002, 0]	[1, 2]	[−0.002, 0]	-

Supplementary Table 2: μ and σ_v priors used to fit the variable Na I absorption line components, where μ is the centre wavelength of the line in nm, and σ_v is the Gaussian line width in km/s. Components correspond to different variable absorption lines present in the same epoch, numbered from 1 (blue-most) to 4 (red-most). This table is also provided in electronic form (see source data in Data Availability in the main text).

	component 1		component 2		component 3		component 4	
Date	μ	σ_v	μ	σ_v	μ	σ_v	μ	σ_v
2018-03-29 (1)	[588.990, 589.000]	[1, 8]	[589.033, 589.043]	[1, 8]	[589.090, 589.100]	[1, 8]	-	-
2018-03-29 (2)	[588.990, 589.000]	[1, 8]	[589.033, 589.043]	[1, 8]	[589.090, 589.100]	[1, 8]	-	-
2018-03-29 (3)	[588.990, 589.000]	[1, 8]	[589.033, 589.043]	[1, 8]	[589.090, 589.100]	[1, 8]	-	-
2018-03-30 (1)	[589.038, 589.042]	[1, 8]	[589.063, 589.073]	[1, 8]	-	-	-	-
2018-03-30 (2)	[589.040, 589.044]	[1, 8]	[589.063, 589.073]	[1, 8]	-	-	-	-
2018-03-30 (3)	[589.043, 589.047]	[1, 8]	[589.063, 589.073]	[1, 8]	-	-	-	-
2018-03-31 (1)	[589.035, 589.045]	[1, 10]	[589.075, 589.085]	[1, 8]	-	-	-	-
2018-03-31 (2)	[589.035, 589.045]	[1, 8]	[589.075, 589.085]	[1, 8]	-	-	-	-
2018-03-31 (3)	[589.030, 589.040]	[1, 10]	[589.075, 589.085]	[1, 8]	-	-	-	-
2018-04-18	[589.085, 589.105]	[1, 8]	-	-	-	-	-	-
2018-04-19	[589.095, 589.105]	[1, 3]	-	-	-	-	-	-
2018-04-20	[589.057, 589.067]	[1, 8]	[589.068, 589.088]	[1, 8]	-	-	-	-
2018-04-21	[588.928, 588.948]	[1, 8]	[589.036, 589.056]	[1, 5]	[589.068, 589.088]	[1, 3]	[589.098, 589.118]	[1, 2]
2018-04-22	[589.035, 589.045]	[1, 8]	[589.053, 589.073]	[1, 8]	[589.095, 589.105]	[1, 3]	-	-
2018-04-23	[589.019, 589.029]	[1, 5]	[589.048, 589.068]	[1, 5]	[589.080, 589.100]	[1, 5]	-	-
2018-05-01	[589.092, 589.102]	[0.1, 5]	-	-	-	-	-	-
2018-05-06	[588.945, 588.955]	[1, 5]	[588.993, 589.003]	[1, 5]	[589.029, 589.039]	[1, 8]	[589.095, 589.105]	[1, 15]
2018-05-13	[589.028, 589.032]	[1, 8]	[589.048, 589.052]	[1, 5]	[589.073, 589.077]	[1, 3]	-	-

Supplementary Table 3: Best fit median values found for the stellar photospheric Na I absorption line, the stationary emission line, and the partially blended stationary Na I absorption lines, where "1" stands for the blue-most feature and "2" stands for the red-most feature. v is the radial velocity in km/s with respect to the stellar rest frame, σ_v is the Gaussian line width in km/s, γ_v is the Lorentzian line width in km/s, τ is the optical depth, f is the surface fill fraction, and $N_{\rm col}$ is the column density in cm^{-2}, calculated from the posterior distribution. Uncertainties correspond to the difference between the median value and the 16 and 84 percentiles of the posterior distribution. This table is also provided in electronic form (see source data in Data Availability in the main text).

	photospheric absorption					stationary emission			
Date	v	σ_v	γ_v	τ	$N_{\rm col}\,(\times10^{12})$	v	σ_v	τ	$N_{\rm col}\,(\times10^{12})$
2018-03-29 (1)	$0.83^{+0.14}_{-0.30}$	$16.65^{+1.25}_{-1.30}$	$24.45^{+1.25}_{-1.25}$	$3.18^{+0.05}_{-0.05}$	$32.39^{+0.54}_{-0.53}$	$0.79^{+0.17}_{-0.37}$	$39.90^{+0.08}_{-0.17}$	$0.77^{+0.01}_{-0.01}$	$7.68^{+0.08}_{-0.08}$
2018-03-29 (2)	$0.96^{+0.04}_{-0.09}$	$17.24^{+1.19}_{-1.19}$	$23.66^{+1.15}_{-1.18}$	$3.11^{+0.05}_{-0.05}$	$31.43^{+0.50}_{-0.50}$	$-0.24^{+0.74}_{-0.55}$	$39.90^{+0.08}_{-0.17}$	$0.78^{+0.01}_{-0.01}$	$7.82^{+0.08}_{-0.08}$
2018-03-29 (3)	$0.98^{+0.03}_{-0.07}$	$19.13^{+0.38}_{-0.47}$	$20.37^{+0.50}_{-0.27}$	$3.00^{+0.04}_{-0.04}$	$29.24^{+0.35}_{-0.34}$	$-0.87^{+0.23}_{-0.11}$	$39.86^{+0.10}_{-0.23}$	$0.79^{+0.01}_{-0.01}$	$7.84^{+0.07}_{-0.07}$
2018-03-30 (1)	$0.99^{+0.02}_{-0.05}$	$19.81^{+0.14}_{-0.31}$	$32.78^{+0.77}_{-0.81}$	$2.83^{+0.05}_{-0.05}$	$36.90^{+0.58}_{-0.63}$	$-0.51^{+0.42}_{-0.32}$	$39.58^{+0.31}_{-0.65}$	$0.85^{+0.01}_{-0.01}$	$8.36^{+0.13}_{-0.15}$
2018-03-30 (2)	$1.00^{+0.01}_{-0.03}$	$19.80^{+0.15}_{-0.33}$	$30.14^{+1.37}_{-1.12}$	$2.94^{+0.06}_{-0.06}$	$36.27^{+1.32}_{-1.06}$	$0.51^{+0.38}_{-0.76}$	$37.09^{+1.71}_{-1.11}$	$0.89^{+0.01}_{-0.01}$	$8.23^{+0.34}_{-0.25}$
2018-03-30 (3)	$1.01^{+0.01}_{-0.02}$	$19.78^{+0.16}_{-0.36}$	$29.62^{+0.82}_{-0.81}$	$2.69^{+0.06}_{-0.05}$	$32.77^{+0.72}_{-0.70}$	$0.86^{+0.12}_{-0.25}$	$33.04^{+0.63}_{-0.57}$	$0.90^{+0.01}_{-0.01}$	$7.46^{+0.16}_{-0.16}$
2018-03-31 (1)	$-0.98^{+0.07}_{-0.03}$	$19.82^{+0.14}_{-0.29}$	$24.94^{+0.43}_{-0.38}$	$3.28^{+0.04}_{-0.03}$	$36.21^{+0.32}_{-0.32}$	$0.81^{+0.15}_{-0.25}$	$39.91^{+0.07}_{-0.15}$	$0.75^{+0.01}_{-0.01}$	$7.51^{+0.05}_{-0.06}$
2018-03-31 (2)	$-0.97^{+0.07}_{-0.03}$	$19.83^{+0.13}_{-0.27}$	$27.23^{+0.42}_{-0.38}$	$3.22^{+0.03}_{-0.03}$	$37.34^{+0.30}_{-0.30}$	$0.90^{+0.09}_{-0.17}$	$39.95^{+0.04}_{-0.09}$	$0.75^{+0.00}_{-0.01}$	$7.50^{+0.05}_{-0.05}$
2018-03-31 (3)	$-0.94^{+0.11}_{-0.06}$	$19.88^{+0.09}_{-0.20}$	$25.47^{+0.36}_{-0.35}$	$3.18^{+0.03}_{-0.03}$	$35.55^{+0.29}_{-0.29}$	$0.94^{+0.06}_{-0.12}$	$39.93^{+0.05}_{-0.11}$	$0.74^{+0.01}_{-0.01}$	$7.41^{+0.05}_{-0.05}$
2018-04-18	$-0.47^{+0.18}_{-0.18}$	$8.71^{+1.40}_{-1.70}$	$29.19^{+0.89}_{-0.86}$	$3.83^{+0.05}_{-0.05}$	$37.55^{+0.37}_{-0.38}$	$0.92^{+0.07}_{-0.16}$	$39.92^{+0.06}_{-0.14}$	$0.61^{+0.00}_{-0.00}$	$6.12^{+0.05}_{-0.05}$
2018-04-19	$-0.01^{+0.21}_{-0.22}$	$19.56^{+0.31}_{-0.57}$	$21.15^{+0.75}_{-0.61}$	$3.73^{+0.07}_{-0.06}$	$37.38^{+0.55}_{-0.56}$	$0.93^{+0.07}_{-0.14}$	$39.51^{+0.36}_{-0.68}$	$0.68^{+0.01}_{-0.01}$	$6.68^{+0.10}_{-0.12}$
2018-04-20	$0.91^{+0.07}_{-0.11}$	$19.90^{+0.08}_{-0.16}$	$20.11^{+0.16}_{-0.08}$	$2.70^{+0.03}_{-0.03}$	$26.54^{+0.29}_{-0.30}$	$0.99^{+0.02}_{-0.04}$	$34.15^{+0.30}_{-0.32}$	$0.87^{+0.00}_{-0.00}$	$7.47^{+0.09}_{-0.09}$
2018-04-21	$-1.00^{+0.02}_{-0.01}$	$11.48^{+1.23}_{-1.37}$	$29.81^{+0.99}_{-0.99}$	$3.72^{+0.05}_{-0.05}$	$39.03^{+0.44}_{-0.46}$	$0.99^{+0.02}_{-0.04}$	$39.94^{+0.04}_{-0.09}$	$0.67^{+0.01}_{-0.01}$	$6.66^{+0.05}_{-0.05}$
2018-04-22	$-0.09^{+0.24}_{-0.23}$	$19.07^{+0.64}_{-0.99}$	$25.10^{+1.03}_{-0.73}$	$3.29^{+0.04}_{-0.04}$	$35.85^{+0.42}_{-0.40}$	$0.89^{+0.10}_{-0.21}$	$39.92^{+0.06}_{-0.12}$	$0.71^{+0.01}_{-0.01}$	$7.12^{+0.06}_{-0.06}$
2018-04-23	$0.94^{+0.06}_{-0.11}$	$19.18^{+0.57}_{-0.84}$	$23.55^{+0.91}_{-0.69}$	$3.25^{+0.04}_{-0.04}$	$34.21^{+0.46}_{-0.44}$	$0.93^{+0.06}_{-0.13}$	$39.83^{+0.13}_{-0.27}$	$0.77^{+0.01}_{-0.01}$	$7.66^{+0.07}_{-0.08}$
2018-05-01	$0.37^{+0.21}_{-0.27}$	$6.80^{+2.06}_{-2.77}$	$34.26^{+0.97}_{-1.05}$	$3.54^{+0.05}_{-0.05}$	$38.91^{+0.42}_{-0.44}$	$0.67^{+0.25}_{-0.47}$	$39.92^{+0.06}_{-0.13}$	$0.72^{+0.01}_{-0.01}$	$7.23^{+0.06}_{-0.06}$
2018-05-06	$0.93^{+0.06}_{-0.10}$	$12.70^{+0.84}_{-0.89}$	$29.14^{+0.76}_{-0.78}$	$3.38^{+0.03}_{-0.03}$	$35.66^{+0.34}_{-0.35}$	$-0.96^{+0.09}_{-0.04}$	$39.88^{+0.09}_{-0.19}$	$0.86^{+0.00}_{-0.00}$	$8.54^{+0.05}_{-0.05}$
2018-05-13	$-0.41^{+0.21}_{-0.23}$	$19.92^{+0.06}_{-0.13}$	$20.12^{+0.18}_{-0.09}$	$3.13^{+0.03}_{-0.03}$	$30.89^{+0.34}_{-0.33}$	$0.79^{+0.16}_{-0.27}$	$34.18^{+0.41}_{-0.40}$	$0.84^{+0.00}_{-0.01}$	$7.15^{+0.10}_{-0.10}$

	stationary absorption 1					stationary absorption 2				
Date	v	σ_v	τ	f	$N_{\rm col}\,(\times10^{12})$	v	σ_v	τ	f	$N_{\rm col}\,(\times10^{12})$
2018-03-29 (1)	$-21.09^{+0.05}_{-0.09}$	$2.58^{+0.18}_{-0.17}$	$0.82^{+0.13}_{-0.08}$	$0.93^{+0.05}_{-0.09}$	$0.53^{+0.09}_{-0.06}$	$-14.98^{+0.04}_{-0.06}$	$2.99^{+0.01}_{-0.02}$	$2.91^{+0.13}_{-0.13}$	$0.86^{+0.01}_{-0.01}$	$2.17^{+0.09}_{-0.09}$
2018-03-29 (2)	$-21.04^{+0.01}_{-0.02}$	$2.13^{+0.13}_{-0.13}$	$1.39^{+0.44}_{-0.37}$	$0.65^{+0.13}_{-0.09}$	$0.74^{+0.20}_{-0.18}$	$-15.33^{+0.06}_{-0.06}$	$3.00^{+0.00}_{-0.01}$	$2.50^{+0.11}_{-0.11}$	$0.91^{+0.02}_{-0.01}$	$1.87^{+0.08}_{-0.08}$
2018-03-29 (3)	$-21.55^{+0.10}_{-0.09}$	$1.43^{+0.12}_{-0.10}$	$0.97^{+0.10}_{-0.07}$	$0.95^{+0.03}_{-0.06}$	$0.35^{+0.05}_{-0.04}$	$-15.48^{+0.07}_{-0.07}$	$3.00^{+0.00}_{-0.01}$	$2.92^{+0.12}_{-0.12}$	$0.89^{+0.01}_{-0.01}$	$2.18^{+0.09}_{-0.09}$
2018-03-30 (1)	$-21.12^{+0.06}_{-0.09}$	$2.70^{+0.11}_{-0.11}$	$1.68^{+0.25}_{-0.23}$	$0.83^{+0.06}_{-0.05}$	$1.14^{+0.15}_{-0.15}$	$-14.93^{+0.00}_{-0.01}$	$3.00^{+0.00}_{-0.00}$	$2.96^{+0.14}_{-0.14}$	$0.90^{+0.01}_{-0.01}$	$2.22^{+0.11}_{-0.10}$
2018-03-30 (2)	$-21.13^{+0.06}_{-0.09}$	$2.25^{+0.11}_{-0.11}$	$1.47^{+0.22}_{-0.16}$	$0.91^{+0.06}_{-0.06}$	$0.83^{+0.11}_{-0.08}$	$-14.93^{+0.01}_{-0.02}$	$3.00^{+0.00}_{-0.01}$	$3.01^{+0.17}_{-0.16}$	$0.86^{+0.02}_{-0.02}$	$2.26^{+0.12}_{-0.12}$
2018-03-30 (3)	$-21.36^{+0.10}_{-0.10}$	$2.14^{+0.10}_{-0.10}$	$1.31^{+0.12}_{-0.07}$	$0.97^{+0.03}_{-0.04}$	$0.71^{+0.06}_{-0.05}$	$-14.93^{+0.01}_{-0.02}$	$3.00^{+0.00}_{-0.01}$	$3.68^{+0.19}_{-0.18}$	$0.83^{+0.01}_{-0.01}$	$2.75^{+0.15}_{-0.13}$
2018-03-31 (1)	$-21.67^{+0.07}_{-0.07}$	$2.05^{+0.07}_{-0.07}$	$1.06^{+0.11}_{-0.06}$	$0.95^{+0.03}_{-0.06}$	$0.54^{+0.05}_{-0.03}$	$-14.94^{+0.01}_{-0.02}$	$3.00^{+0.00}_{-0.01}$	$2.64^{+0.11}_{-0.11}$	$0.86^{+0.01}_{-0.01}$	$1.98^{+0.08}_{-0.08}$
2018-03-31 (2)	$-21.48^{+0.10}_{-0.11}$	$2.28^{+0.09}_{-0.10}$	$0.98^{+0.05}_{-0.03}$	$0.98^{+0.02}_{-0.03}$	$0.56^{+0.03}_{-0.03}$	$-14.97^{+0.04}_{-0.06}$	$3.00^{+0.00}_{-0.01}$	$2.12^{+0.10}_{-0.09}$	$0.92^{+0.02}_{-0.02}$	$1.59^{+0.07}_{-0.07}$
2018-03-31 (3)	$-21.90^{+0.09}_{-0.08}$	$2.04^{+0.10}_{-0.09}$	$0.98^{+0.04}_{-0.03}$	$0.98^{+0.01}_{-0.02}$	$0.50^{+0.03}_{-0.02}$	$-15.07^{+0.06}_{-0.06}$	$3.00^{+0.00}_{-0.01}$	$2.14^{+0.08}_{-0.08}$	$0.96^{+0.01}_{-0.01}$	$1.60^{+0.06}_{-0.06}$
2018-04-18	$-22.54^{+0.11}_{-0.11}$	$1.46^{+0.12}_{-0.11}$	$0.72^{+0.14}_{-0.08}$	$0.91^{+0.07}_{-0.11}$	$0.27^{+0.05}_{-0.04}$	$-15.38^{+0.07}_{-0.07}$	$2.97^{+0.02}_{-0.04}$	$1.71^{+0.09}_{-0.06}$	$0.98^{+0.02}_{-0.02}$	$1.27^{+0.06}_{-0.04}$
2018-04-19	$-22.52^{+0.10}_{-0.10}$	$1.01^{+0.10}_{-0.09}$	$2.56^{+1.04}_{-0.82}$	$0.59^{+0.10}_{-0.05}$	$0.64^{+0.19}_{-0.17}$	$-15.50^{+0.07}_{-0.07}$	$2.35^{+0.08}_{-0.08}$	$1.73^{+0.08}_{-0.06}$	$0.99^{+0.01}_{-0.01}$	$1.02^{+0.04}_{-0.03}$
2018-04-20	$-23.00^{+0.05}_{-0.04}$	$1.87^{+0.08}_{-0.07}$	$0.80^{+0.11}_{-0.06}$	$0.93^{+0.05}_{-0.08}$	$0.37^{+0.05}_{-0.03}$	$-15.38^{+0.04}_{-0.04}$	$2.98^{+0.01}_{-0.02}$	$1.59^{+0.03}_{-0.02}$	$0.99^{+0.00}_{-0.01}$	$1.18^{+0.02}_{-0.02}$
2018-04-21	$-22.55^{+0.16}_{-0.25}$	$1.27^{+0.16}_{-0.11}$	$3.89^{+0.55}_{-0.49}$	$0.66^{+0.03}_{-0.03}$	$1.24^{+0.16}_{-0.14}$	$-15.90^{+0.08}_{-0.08}$	$2.94^{+0.04}_{-0.06}$	$1.54^{+0.05}_{-0.04}$	$0.99^{+0.01}_{-0.01}$	$1.13^{+0.03}_{-0.03}$
2018-04-22	$-22.50^{+0.12}_{-0.12}$	$1.62^{+0.11}_{-0.10}$	$1.28^{+0.30}_{-0.21}$	$0.84^{+0.10}_{-0.09}$	$0.52^{+0.11}_{-0.08}$	$-16.10^{+0.08}_{-0.08}$	$2.71^{+0.08}_{-0.08}$	$1.68^{+0.11}_{-0.08}$	$0.97^{+0.02}_{-0.03}$	$1.14^{+0.07}_{-0.05}$
2018-04-23	$-23.02^{+0.06}_{-0.03}$	$2.17^{+0.13}_{-0.12}$	$1.34^{+0.31}_{-0.28}$	$0.73^{+0.11}_{-0.08}$	$0.73^{+0.14}_{-0.13}$	$-15.95^{+0.07}_{-0.07}$	$2.89^{+0.07}_{-0.08}$	$1.72^{+0.12}_{-0.09}$	$0.96^{+0.02}_{-0.03}$	$1.24^{+0.07}_{-0.06}$
2018-05-01	$-22.20^{+0.12}_{-0.13}$	$1.19^{+0.10}_{-0.09}$	$3.95^{+0.67}_{-0.71}$	$0.58^{+0.05}_{-0.04}$	$1.17^{+0.17}_{-0.19}$	$-16.90^{+0.07}_{-0.04}$	$2.92^{+0.05}_{-0.07}$	$2.06^{+0.16}_{-0.15}$	$0.93^{+0.03}_{-0.03}$	$1.51^{+0.11}_{-0.10}$
2018-05-06	$-22.74^{+0.08}_{-0.08}$	$1.66^{+0.09}_{-0.09}$	$0.79^{+0.24}_{-0.16}$	$0.79^{+0.14}_{-0.13}$	$0.33^{+0.09}_{-0.06}$	$-16.07^{+0.05}_{-0.05}$	$2.47^{+0.06}_{-0.06}$	$1.13^{+0.03}_{-0.02}$	$0.99^{+0.01}_{-0.01}$	$0.70^{+0.02}_{-0.02}$
2018-05-13	$-22.39^{+0.07}_{-0.07}$	$1.74^{+0.09}_{-0.08}$	$1.20^{+0.27}_{-0.25}$	$0.73^{+0.11}_{-0.08}$	$0.52^{+0.10}_{-0.09}$	$-15.81^{+0.05}_{-0.05}$	$2.24^{+0.06}_{-0.06}$	$1.22^{+0.03}_{-0.03}$	$0.99^{+0.01}_{-0.01}$	$0.69^{+0.02}_{-0.02}$

Supplementary Table 4: Best fit median values found for the variable Na I absorption lines. v is the radial velocity in km/s with respect to the stellar rest frame, σ_v is the Gaussian line width in km/s, τ is the optical depth, f is the surface fill fraction, and $N_{\rm col}$ is the column density in cm^{-2}, calculated from the posterior distribution. Uncertainties correspond to the difference between the median value and the 16 and 84 percentiles of the posterior distribution. This table is also provided in electronic form (see source data in Data Availability in the main text).

	component 1					component 2				
Date	v	σ_v	τ	f	$N_{\rm col}\,(\times 10^{12})$	v	σ_v	τ	f	$N_{\rm col}\,(\times 10^{12})$
2018-03-29 (1)	$-80.93^{+0.23}_{-0.28}$	$6.08^{+0.17}_{-0.17}$	$3.83^{+0.37}_{-0.33}$	$0.40^{+0.01}_{-0.01}$	$5.83^{+0.49}_{-0.45}$	$-59.50^{+0.16}_{-0.18}$	$6.05^{+0.15}_{-0.14}$	$2.57^{+0.15}_{-0.14}$	$0.65^{+0.01}_{-0.01}$	$3.90^{+0.20}_{-0.20}$
2018-03-29 (2)	$-80.91^{+0.21}_{-0.24}$	$6.21^{+0.16}_{-0.16}$	$2.60^{+0.20}_{-0.19}$	$0.47^{+0.01}_{-0.01}$	$4.03^{+0.28}_{-0.28}$	$-59.40^{+0.15}_{-0.17}$	$6.33^{+0.16}_{-0.14}$	$2.44^{+0.13}_{-0.13}$	$0.66^{+0.01}_{-0.01}$	$3.87^{+0.18}_{-0.17}$
2018-03-29 (3)	$-80.78^{+0.14}_{-0.18}$	$5.75^{+0.12}_{-0.13}$	$2.75^{+0.17}_{-0.16}$	$0.53^{+0.01}_{-0.01}$	$3.95^{+0.20}_{-0.20}$	$-59.60^{+0.12}_{-0.14}$	$6.66^{+0.14}_{-0.13}$	$1.95^{+0.11}_{-0.10}$	$0.73^{+0.02}_{-0.02}$	$3.25^{+0.15}_{-0.15}$
2018-03-30 (1)	$-60.81^{+0.26}_{-0.23}$	$2.83^{+0.12}_{-0.11}$	$4.62^{+0.28}_{-0.49}$	$0.30^{+0.02}_{-0.01}$	$3.24^{+0.19}_{-0.28}$	$-46.76^{+0.17}_{-0.16}$	$4.99^{+0.11}_{-0.12}$	$4.96^{+0.03}_{-0.06}$	$0.62^{+0.01}_{-0.01}$	$6.17^{+0.15}_{-0.16}$
2018-03-30 (2)	$-59.03^{+0.37}_{-0.35}$	$3.09^{+0.21}_{-0.17}$	$3.41^{+0.98}_{-0.89}$	$0.31^{+0.04}_{-0.02}$	$2.64^{+0.60}_{-0.61}$	$-45.40^{+0.13}_{-0.13}$	$3.99^{+0.09}_{-0.08}$	$4.97^{+0.02}_{-0.05}$	$0.71^{+0.01}_{-0.01}$	$4.94^{+0.12}_{-0.11}$
2018-03-30 (3)	$-57.32^{+0.29}_{-0.30}$	$3.25^{+0.37}_{-0.38}$	$0.72^{+0.56}_{-0.27}$	$0.57^{+0.25}_{-0.18}$	$0.59^{+0.37}_{-0.21}$	$-44.33^{+0.12}_{-0.11}$	$4.06^{+0.07}_{-0.07}$	$4.98^{+0.01}_{-0.03}$	$0.73^{+0.01}_{-0.01}$	$5.04^{+0.09}_{-0.09}$
2018-03-31 (1)	$-57.69^{+0.02}_{-0.04}$	$9.92^{+0.06}_{-0.10}$	$2.86^{+0.12}_{-0.11}$	$0.54^{+0.01}_{-0.01}$	$7.07^{+0.28}_{-0.26}$	$-39.40^{+0.07}_{-0.07}$	$4.28^{+0.05}_{-0.05}$	$4.97^{+0.03}_{-0.05}$	$0.71^{+0.00}_{-0.00}$	$5.30^{+0.06}_{-0.07}$
2018-03-31 (2)	$-60.80^{+0.16}_{-0.16}$	$6.62^{+0.14}_{-0.14}$	$2.63^{+0.11}_{-0.10}$	$0.69^{+0.01}_{-0.01}$	$4.35^{+0.14}_{-0.14}$	$-41.25^{+0.12}_{-0.12}$	$4.84^{+0.06}_{-0.06}$	$4.96^{+0.03}_{-0.06}$	$0.70^{+0.00}_{-0.00}$	$5.99^{+0.08}_{-0.09}$
2018-03-31 (3)	$-61.44^{+0.16}_{-0.15}$	$7.08^{+0.12}_{-0.12}$	$2.63^{+0.09}_{-0.08}$	$0.72^{+0.01}_{-0.01}$	$4.65^{+0.14}_{-0.14}$	$-41.75^{+0.11}_{-0.11}$	$4.77^{+0.08}_{-0.08}$	$4.56^{+0.17}_{-0.17}$	$0.70^{+0.01}_{-0.01}$	$5.45^{+0.16}_{-0.16}$
2018-04-18	$-31.64^{+0.19}_{-0.19}$	$5.31^{+0.17}_{-0.16}$	$2.26^{+0.18}_{-0.17}$	$0.68^{+0.02}_{-0.02}$	$2.99^{+0.22}_{-0.21}$	-	-	-	-	-
2018-04-19	$-31.83^{+0.17}_{-0.16}$	$2.99^{+0.01}_{-0.01}$	$4.91^{+0.06}_{-0.14}$	$0.45^{+0.01}_{-0.01}$	$3.67^{+0.05}_{-0.11}$	-	-	-	-	-
2018-04-20	$-48.34^{+0.18}_{-0.18}$	$2.57^{+0.12}_{-0.11}$	$3.43^{+0.51}_{-0.42}$	$0.32^{+0.02}_{-0.02}$	$2.21^{+0.26}_{-0.22}$	$-36.74^{+0.10}_{-0.10}$	$4.46^{+0.11}_{-0.10}$	$2.58^{+0.11}_{-0.11}$	$0.70^{+0.01}_{-0.01}$	$2.88^{+0.10}_{-0.10}$
2018-04-21	$-111.59^{+0.20}_{-0.19}$	$6.98^{+0.27}_{-0.28}$	$0.57^{+0.23}_{-0.14}$	$0.70^{+0.19}_{-0.16}$	$1.00^{+0.35}_{-0.24}$	$-53.84^{+0.16}_{-0.16}$	$4.99^{+0.01}_{-0.02}$	$1.98^{+0.20}_{-0.19}$	$0.51^{+0.03}_{-0.02}$	$2.47^{+0.25}_{-0.24}$
2018-04-22	$-58.63^{+0.43}_{-0.37}$	$3.92^{+0.46}_{-0.42}$	$0.74^{+0.75}_{-0.40}$	$0.38^{+0.33}_{-0.14}$	$0.74^{+0.61}_{-0.37}$	$-44.73^{+0.08}_{-0.08}$	$4.02^{+0.05}_{-0.05}$	$4.88^{+0.08}_{-0.13}$	$0.81^{+0.00}_{-0.00}$	$4.89^{+0.09}_{-0.10}$
2018-04-23	$-65.85^{+0.03}_{-0.07}$	$4.59^{+0.25}_{-0.29}$	$3.87^{+0.75}_{-0.96}$	$0.11^{+0.01}_{-0.01}$	$4.39^{+0.82}_{-1.04}$	$-48.34^{+0.06}_{-0.06}$	$2.72^{+0.03}_{-0.04}$	$4.96^{+0.03}_{-0.07}$	$0.85^{+0.00}_{-0.00}$	$3.37^{+0.05}_{-0.05}$
2018-05-01	$-28.88^{+0.14}_{-0.18}$	$3.28^{+0.10}_{-0.11}$	$4.44^{+0.28}_{-0.26}$	$0.76^{+0.01}_{-0.01}$	$3.63^{+0.22}_{-0.22}$	-	-	-	-	-
2018-05-06	$-106.05^{+0.16}_{-0.16}$	$4.97^{+0.02}_{-0.04}$	$1.25^{+0.23}_{-0.22}$	$0.29^{+0.04}_{-0.03}$	$1.56^{+0.28}_{-0.27}$	$-82.25^{+0.37}_{-0.38}$	$4.97^{+0.02}_{-0.04}$	$0.23^{+0.13}_{-0.05}$	$0.72^{+0.20}_{-0.24}$	$0.28^{+0.16}_{-0.06}$
2018-05-13	$-66.30^{+0.02}_{-0.01}$	$7.03^{+0.08}_{-0.08}$	$2.69^{+0.10}_{-0.10}$	$0.72^{+0.01}_{-0.01}$	$4.73^{+0.15}_{-0.14}$	$-54.11^{+0.00}_{-0.01}$	$3.02^{+0.07}_{-0.07}$	$3.75^{+0.30}_{-0.27}$	$0.59^{+0.01}_{-0.01}$	$2.84^{+0.18}_{-0.17}$

	component 3					component 4				
Date	v	σ_v	τ	f	$N_{\rm col}\,(\times 10^{12})$	v	σ_v	τ	f	$N_{\rm col}\,(\times 10^{12})$
2018-03-29 (1)	$-29.96^{+0.20}_{-0.33}$	$5.43^{+0.32}_{-0.35}$	$1.51^{+0.22}_{-0.21}$	$0.59^{+0.05}_{-0.04}$	$2.05^{+0.28}_{-0.29}$	-	-	-	-	-
2018-03-29 (2)	$-29.81^{+0.10}_{-0.21}$	$5.68^{+0.28}_{-0.32}$	$1.96^{+0.23}_{-0.21}$	$0.51^{+0.03}_{-0.02}$	$2.78^{+0.32}_{-0.30}$	-	-	-	-	-
2018-03-29 (3)	$-30.55^{+0.30}_{-0.27}$	$6.00^{+0.30}_{-0.28}$	$1.55^{+0.17}_{-0.17}$	$0.59^{+0.04}_{-0.03}$	$2.33^{+0.24}_{-0.24}$	-	-	-	-	-
2018-03-30 (1)	-	-	-	-	-	-	-	-	-	-
2018-03-30 (2)	-	-	-	-	-	-	-	-	-	-
2018-03-30 (3)	-	-	-	-	-	-	-	-	-	-
2018-03-31 (1)	-	-	-	-	-	-	-	-	-	-
2018-03-31 (2)	-	-	-	-	-	-	-	-	-	-
2018-03-31 (3)	-	-	-	-	-	-	-	-	-	-
2018-04-18	-	-	-	-	-	-	-	-	-	-
2018-04-19	-	-	-	-	-	-	-	-	-	-
2018-04-20	-	-	-	-	-	-	-	-	-	-
2018-04-21	$-38.14^{+0.09}_{-0.09}$	$2.99^{+0.00}_{-0.01}$	$4.97^{+0.03}_{-0.05}$	$0.75^{+0.01}_{-0.01}$	$3.71^{+0.02}_{-0.04}$	$-27.26^{+0.16}_{-0.25}$	$1.99^{+0.01}_{-0.02}$	$4.92^{+0.06}_{-0.14}$	$0.70^{+0.01}_{-0.02}$	$2.44^{+0.04}_{-0.07}$
2018-04-22	$-28.14^{+0.14}_{-0.14}$	$2.99^{+0.01}_{-0.02}$	$3.49^{+0.23}_{-0.22}$	$0.69^{+0.01}_{-0.01}$	$2.60^{+0.17}_{-0.16}$	-	-	-	-	-
2018-04-23	$-37.07^{+0.31}_{-0.35}$	$4.82^{+0.13}_{-0.24}$	$1.12^{+0.34}_{-0.31}$	$0.52^{+0.13}_{-0.08}$	$1.33^{+0.41}_{-0.36}$	-	-	-	-	-
2018-05-01	-	-	-	-	-	-	-	-	-	-
2018-05-06	$-65.01^{+0.58}_{-0.52}$	$5.70^{+0.63}_{-0.59}$	$0.17^{+0.21}_{-0.06}$	$0.57^{+0.29}_{-0.30}$	$0.24^{+0.29}_{-0.08}$	$-27.20^{+0.05}_{-0.10}$	$7.37^{+0.20}_{-0.20}$	$0.55^{+0.12}_{-0.07}$	$0.84^{+0.10}_{-0.12}$	$1.01^{+0.22}_{-0.13}$
2018-05-13	$-43.40^{+0.02}_{-0.01}$	$3.00^{+0.00}_{-0.01}$	$4.89^{+0.08}_{-0.16}$	$0.46^{+0.01}_{-0.01}$	$3.66^{+0.06}_{-0.12}$	-	-	-	-	-

Supplementary Table 5: Input orbital parameters used in N-body simulations with `REBOUND` with planets b and c (2-planet simulations) and b, c, and d (3-planet simulations). Parameters include mass (M) in M_{Jup}, semi-major axis (a) in au, eccentricity (e), inclination relative to the disc inclination (i) in degrees, argument of periastron (ω) in degrees, and longitude of the ascending node (Ω) in degrees, and stellar mass ($M_\star$) in $M_\odot$. Values in this table correspond to the median value of the posterior of the "stable (including N-body)" orbital parameters from Tables 3 and 4 of [2] for the first (2-planet) and second (3-planet), respectively. This table is also provided in electronic form (see source data in Data Availability in the main text).

2-planet simulations	Object	M (M_{Jup})	a (au)	e	i (deg)	ω (deg)	Ω (deg)
	PDS 70 b	1.4	20.7	0.160	2.3	190.0	176.0
	PDS 70 c	6.4	33.9	0.042	1.5	77.0	158.0
	PDS 70			$M_\star = 0.952$ $M_\odot$			

3-planet simulations	Object	M (M_{Jup})	a (au)	e	i (deg)	ω (deg)	Ω (deg)
	PDS 70 b	0.7	21.1	0.131	0.4	191.4	174.3
	PDS 70 c	2.4	35.3	0.033	0.2	63.0	159.8
	PDS 70 d	0.4	10.7	0.250	22.7	29.0	144.0
	PDS 70			$M_\star = 0.965$ $M_\odot$			

References

[1] Donati, J. F. *et al.* Magnetospheric accretion and spin-down of the prototypical classical T Tauri star AA Tau. *MNRAS* **409**, 1347–1361 (2010).

[2] Trevascus, D. *et al.* Differentiating Formation Models with New Dynamical Masses for the PDS 70 Protoplanets. *A&A* **698**, A19 (2025).
