# Supplementary material for: Potential sublimating exocomets around the young star PDS 70
Source: Nat Commun. 2026 Aug 24;17:8894. doi: 10.1038/s41467-026-76880-y (PMC13503679; doi:10.1038/s41467-026-76880-y)
Supplement: Supplementary file 2 [file 41467_2026_76880_MOESM2_ESM.pdf]

nature portfolio

Peer Review File

Potential sublimating exocomets around the young star PDS 70

Corresponding Author: Dr Aline Novais

This file contains all reviewer reports in order by version, followed by all author rebuttals in order by version.

This manuscript has been previously reviewed at another journal. This document only contains information relating to versions considered at Nature Communications.

Version 0:

Reviewer comments:

Reviewer #3

(Remarks to the Author)
The authors have performed a detailed examination of archival HARPS observations of the system PDS 70 and have found variable absorption features they believe are attributable to exocomets in highly elliptical orbits around the star. The presence of exocomets in this system would be of high impact, as no exocomets have been detected before in a K-type star via spectroscopy. In addition, the system is particularly young and hosts two protoplanets. This would advance our understanding on planetary systems formation and evolution.

The authors have done a good job in addressing several aspects to assess the exocomets scenario including comparison with stellar winds, accretion, stellar spots, variability in other T Tauri stars, and simulations of orbital inclinations and efficiency of scattering of exocomets.

However, given the evidence presented in this paper, I do not think these strongly confirm the exocomet scenario. I agree with the last comment of reviewer #1 and also think the results should be significantly toned down. Instead, I'd say that exocomets might be "a possible explanation" or that "the exocomet scenario cannot be ruled out". I do not see this study as conclusive evidence for exocomets and I explain why:

The N-body simulations performed by the authors show that an average of 0.47% of planetesimals are observed to have crossed the gap towards the inner disc (with confirmed protoplanets b and c, and higher percentage if potential planet d was considered). Then, about 10% of these planetesimals would evolve into high orbital inclinations, enough to be transiting the line of sight (or 6% if considering planet d). Yet, the authors found 42 blue-shifted Na I absorption line components which they attribute to exocomets within only 18 epochs of observations. It seems unlikely to be able to detect such high level of exocometary activity (comparable to Beta Pictoris) when the odds of exocomets transiting are quite low. Beta Pic has a particularly favourable orientation (almost exactly edge-on), whereas PDS 70, unfortunately, does not.

Regarding the disc winds, these estimations are model dependant and based on several assumptions. Even the mass accretion rate values for T Tauris can have uncertainties of nearly an order of magnitude (see some of the Manara's papers on accretion rates). So I think the column densities being about an order of magnitude higher than that expected for disc winds is not a strong argument in favour of the exocomet scenario as these values can be within uncertainties and thus compatible with winds.

In the section about the origin of the blue-shifted absorption lines, the authors say that the detections are biased towards the blue-shifted side of the sodium lines because the red-shifted part of the spectrum is strongly contaminated by the stellar accretion emission line. I disagree with this statement. When I look at figure 1b, I see that the accretion line is well centered at the stellar radial velocity, so there should not be any bias towards blue-shifted or red-shifted detections. Although it is true that blue-shifted absorptions are plausible for exocomets transits, the lack of red-shifted absorptions makes me more skeptical about the exocomet scenario, as the presence of only-blueshifted absorptions is consistent with winds.

Also the lack of CaII absorption makes the exocomet scenario less convincing. As the authors mention in their analysis; absorptions are expected in the CaII H&K lines (if produced by exocomets sublimation). In order to prove that the lack of absorption in the UV lines is due to bad SNR, I'd suggest to inject in the spectra the expected signal of an absorption in the CaII K line. Since for exocomets, column densities for CaII are expected to be higher than for NaI (Kiefer et al. 2014),

perhaps it would be a good idea to adopt a detection limit for similar total column densities: N(CaII K+H) >= N(NaI D1+D2) and similar optical depths to NaI, and compare that to the deepest NaI detection to check the detectability in CaII K.

A side concern; the authors' response to reviewer 1 comment regarding the ISM contribution. I believe a significant part of their response is very wrong. The authors claim it is "highly unlikely" for the ISM to create the pseudo-stable absorption feature at -19 km/s and do NOT believe that the observed lines at -19km/s can plausibly be attributed to the ISM. The authors should be aware that the study of Redfield & Linsky (2008) is limited to a distance of 15pc. Any interstellar cloud beyond this distance will not appear in this survey. The LIC is only the Local Insterstellar Cloud, which is the cloud where the Solar System is embedded. The interstellar medium is filled with clouds with a wide range of densities. Since PDS 70 is at a distance of ~112pc (according to Gaia EDR3), then it is very likely that there are more interstellar clouds (beyond the LIC) crossing the line of sight of the star. The more distant the star, the more ISM material can be crossing its line of sight. Examples of ISM absorptions with NaI column densities in the order of 10^12 can be found in Welsh et al. (2010); please look at figures 2 to 5. In some of these examples the line depths exceed 50%. Therefore, I think it is highly plausible that some of the pseudo-stable blue-shifted absorptions found in the spectra are due to ISM contribution. Of course, these do not explain the variable absorption features or any correlation with stellar rotation, but it would be a good idea to assess any possible ISM contribution in the spectra to better account for additional absorptions.

Overall, I think given that the system does not have a favourable orientation, and the lack of CaII detection, and the system being a T Tauri with ongoing accretion and likely to have winds, and only blue-shifted absorptions; exocomets do not seem as the most likely explanation for the variable absorption features detected in NaI. Winds, in my opinion, seem like a more plausible cause. I hope the authors can prove me wrong in the future, as this would be certainly a very interesting discovery, but the evidence presented so far is not strongly convincing. Hopefully, the authors could obtain stronger evidence in future observations such as an asymmetric transit in photometry or simultaneous CaII and NaI detections in spectra with better SNR. I'd suggest trying to obtain UVES or ESPRESSO observations as these could achieve a better SNR than HARPS.

(Remarks on code availability)

Reviewer #4

(Remarks to the Author)
The paper entitled "Sublimating exocomets in the PDS 70 system" by Novais et al. presents the spectroscopic detection of exocomets transiting the young protostar PDS 70.

The results presented in this manuscript are of prime importance, as they propose a plausible and physically consistent scenario for the origin of the water detected by JWST in this well-studied young planetary system. In a broader context where only a handful of exocometary systems have been identified to date, this system represents a particularly valuable new laboratory for the study of exocomets. It is therefore likely to become a prime target for future observations with the most advanced astronomical facilities.

The two previous referees raised several important concerns, notably regarding the inclination of the exocometary orbits, the possibility that the detected spectroscopic absorptions could originate from stellar winds (Referee #1), and the non-detection of Ca II (Referee #2). The authors have now convincingly addressed all these points through the inclusion of new numerical simulations, a detailed quantitative analysis of the stellar wind scenario, and an improved assessment of the signal-to-noise ratio demonstrating that the Ca II non-detection is fully consistent with observational limitations. These additions significantly strengthen the manuscript and make the proposed exocometary interpretation compelling.

I have a few minor comments detailed below :

1) Most important comment :
Several tables listing the adopted priors are provided (Table 3 and subsequent tables), but I could not find any table summarizing the numerical results of the fits to the 42 individual absorption signatures. Figure 3 alone is insufficient for this purpose. A comprehensive table reporting the best-fit parameters (and associated uncertainties) for each absorption event is currently missing in the paper.

2) In the Introduction, when discussing photometric detections of exocomets, it would be appropriate to also cite the recent studies by Pavlenko et al. (2022), Lecavelier Des Etangs et al. (2022), and Kiefer et al. (2023).

3) Line 122.
A citation to the reference [20] in addition to the reference [12] would be useful, as it provides tools to quantify the covering factor (as used in the present work).

4) Line 183.
Converting the inferred sodium mass into a characteristic size of the parent body would be helpful for the reader, for instance by assuming a meteoritic sodium abundance. This would allow a direct assessment of whether the inferred masses are realistic. A rough estimate suggests that a kilometer-sized body could already account for a few 10^9 kg of sodium, but this deserves to be quantified more carefully.

5) Line 185.

The comparison between the measured sodium column density and that reported for comet McNaught (C/2006 P1) appears somewhat hazardous. In the latter case, the sodium measurement was obtained in emission and probes a limited region along the line of sight, making a direct comparison with the present absorption measurements potentially misleading.

6) Line 257.
It seems that the sodium column densities measured in the absorption lines are two, rather than one, orders of magnitude higher than those expected from the stellar wind scenario.

7) Line 905.
It is unclear why the R line ratio is treated as a free parameter in Eq. 1, instead of being fixed to its expected value of 2. A justification for this choice, or a discussion of its impact on the results, would be welcome.

8) Line 1322. Caption of Fig. 3
The observed line broadening likely has a dynamical origin for exocomets located close to their host star. Here, the gas temperature may be largely independent of the distance to the star (see Vrignaud et al. 2025).

(Remarks on code availability)
I verified that the code is publicly available and that all required files appear to be included.

However, I did not attempt to run it or to assess its proper functioning.

Reviewer #5

(Remarks to the Author)
Dear authors,

Thank you for your careful revision and for tackling the concerns raised by the previous reviewers. A few parts of the manuscript still require clarification or a more explicit discussion. Below, the remaining points to clarify from previous referee reports are outlined, along with some additional comments.

I co-reviewed this manuscript with one of the reviewers who provided the listed reports. This is part of the Nature Communications initiative to facilitate training in peer review and to provide appropriate recognition for Early Career Researchers who co-review manuscripts.

Points linked to previous referee reports:

Page 9:
Line 394:
The number of planetesimals scattered to the inner regions would strongly depend on their initial parameters, such as how close they are to the chaotic zone of c, of resonances in the outer belt, their initial eccentricities and inclinations, as well as the presence or absence of gas that can damp the orbits of solids. The text could acknowledge that these fractions are highly dependent on the assumed initial conditions and neglect gas drag, and point the reader to the supplementary material. It would also be interesting, if feasible, to show how the rate changes when varying these parameters.

Line 410:
The quoted 10% rate may depend strongly on the initial inclination distribution given to the planetesimals, which is relatively high if planetesimals are born very close to the midplane, where most large grains are. The authors should either add a caveat here or run additional simulations to demonstrate that the result is robust to changes in the initial conditions.

Page 18:
I agree that any variable absorption features in the CaII H&K lines would be undetectable due to the low SNR of the spectrum at those wavelengths. The calcium doublet is predominantly used to investigate the level of stellar activity in late-type stars, and this activity manifests as a (sometimes variable) emission feature that dominates over the photospheric stellar absorption (e.g., Strassmeier et al. 1990; Bendahan-West et al. 2025). In the case of PDS 70, there is strong CaII H&K emission (visible in the PDS 70 spectra shown as an additional figure in the response to comments of Reviewer#2), which would hinder any possibility of observing variable absorption features due to exocomet transits. I suggest adding a note on this, as this would strengthen the justification for the lack of CaII H&K detections.

Page 23:
"N-body simulations" section:
As mentioned earlier, the results of these simulations may depend strongly on the adopted initial conditions. There are three points that I find concerning:

i) First, how many particles are scattered inwards will strongly depend on how close those particles are to the outermost planet (PDS 70 c). If those particles are placed beyond ~3 Hill radii or the chaotic zone (e.g. Pearce et al. 2014, Marino et al. 2018), they may never be excited to an eccentric orbit to later be scattered by planet c. So, to make these results transparent,

the authors could include a 5th and 6th panel in Figure 7 showing eccentricity vs semi-major axis for all particles at the end of their simulation, marking the minimum and maximum initial semi-major axes, and in addition, add the initial semi-major axis and eccentricities in colour of those that cross the gap (or as a density distribution as a function of semi-major axis as they start with zero eccentricity). This will determine whether the particles that cross the gap start all very close to the inner edge of the outer disc or not. If so, the authors should add a sentence stating that the inferred fraction strongly depends on the assumed initial semi-major axis of planetesimals.

ii) I suggest that the authors examine how the results would vary if they assumed smaller initial inclinations (or even set them to zero) and justify using initial inclinations between ±10 deg. The initial inclination distribution should affect the calculated rates of planetesimals crossing the gap with sufficiently high inclinations, and these rates would likely be lower for smaller initial inclinations. This is because the higher the inclination, the more likely particles are to get a vertical kick by a planet that raises the inclination even higher. Since planetesimals may be born very close to the midplane, starting inclinations with ±10deg may significantly inflate the number of particles that cross the gap with 40 deg inclinations. The authors may not need to run additional simulations, and instead they could split their particles between those that start with low inclination (e.g. <1 deg) and those that start with high inclination (e.g >1 deg) to assess this.

iii) In addition, the simulations do not include any gas, which would damp planetesimal inclinations and thus could modify the inferred rates (e.g. Raymond et al, 2017). This damping may favour inward scattering relative to ejection, but it may disfavour the excitement of inclinations above 40 deg. I ask the authors to include this caveat in the text.

Page 27:
Fig 1b:
The “disc lines” would benefit from a more explicit discussion in the paper, particularly regarding their variability with the stellar rotation period and the possible ISM contribution (as explained in the responses to comments from Reviewer#1). Even if the feature is not attributed to the ISM alone, the authors should still discuss the potential ISM contribution in the text.

Additional comments:

Page 2:
Line 77: The authors could quantify “very short timescales” to indicate the typical variability timescale associated with exocomet transits.

Page 4:
Line 148-149:
The discussion of the surface fill fraction needs a caveat. The calculation appears to assume that the entire gas cloud is projected on the stellar disc. However, it is possible that the cloud only grazes the stellar disc. Because the surface fill fraction is a key parameter used to support an exocometary origin, this caveat and assumption should be stated, as it may affect some of the results and conclusions.

Line 179-180:
It is not clear why the analysis assumes optically thin lines, given that the optical depths are known and many of the lines are optically thick. On this line, “some” could be replaced by “many” to reflect the fact that a substantial fraction of the lines are optically thick (based on Fig. 3b).

Line 272:
Should A = f*pi*Rstar^2 be used instead of f*pi*(2*Rstar)^2? This change would lower the inferred mass loss rates and bring them closer to estimates from disc winds.

Page 8:
“Origin of the blue-shifted sodium lines” section:
The fact that all variable features are blue-shifted is interesting dynamically. It would be useful to include a comparison with the predominantly red-shifted features seen in the archetypal system Beta Pictoris, especially since much of the exocomet literature is built around that system.

In addition, line 370 suggests that the red-shifted part of the spectrum is strongly contaminated by the stellar accretion emission line, hindering the observation of red-shifted features. However, Fig. 1b indicates that the accretion emission only affects low-velocity features, so the implications for the lack of higher-velocity red-shifted components could be discussed more explicitly.

Page 32:
Fig. 6:
It is unclear how the numbers in the figure are assigned; please clarify in the caption.

Page 33:
Fig. 7b-d:
The paper would benefit from an equivalent figure with eccentricity vs semimajor axis for these simulations. Additionally, it could be helpful to overplot in grey the orbital parameters of the planetesimals that survive at the end of the 5Myr as suggested in one of my earlier comments.

Typos:

Page 6:
Line 249:
"...as being a fraction the mass…" → "...as being a fraction of the mass…"

(Remarks on code availability)

Reviewer #6

(Remarks to the Author)
I co-reviewed this manuscript with one of the reviewers who provided the listed reports. This is part of the Nature Communications initiative to facilitate training in peer review and to provide appropriate recognition for Early Career Researchers who co-review manuscripts.

(Remarks on code availability)

Version 1:

Reviewer comments:

Reviewer #3

(Remarks to the Author)
The PDS 70 system is a young T Tauri star surrounded by a transition disc of gas and dust with ongoing planet formation as evidenced by the detection of two protoplanets. The system also shows the presence of water in the inner regions of the disc in JWST observations. In the present study the authors have found variable blue-shifted absorption lines of neutral sodium in HARPS spectra of the PDS 70 system. The author performed a detailed analysis of these lines and assessed several hyphotheses to explain the most likely origin of these sodium absorptions.The strongest contenders are disc winds and sublimating exocomets, and the authors conclude that exocomets on highly elliptical orbits would be the most likely scenario. This possibility could be connected to water delivery to the inner region of this system and therefore provide significant insight on the transport of water towards rocky planet during early stages of planet formation.

I see that the authors have fully taken into account the comments provided by the reviewers and have carefully addressed all concerns. The paper has been significantly improved, analysis and results are presented cautiously and meticulously, addressing sources of uncertainty, limitations and caveats, which overall paints a clearer picture on the plausibility of each scenario. I am satisfied with this version of the paper, the way it is now presented, and the answers to the referees comments. I now see the exocomet scenario as more plausible under the evidence of day-to-day strong variability being uncommon in disc winds. Furthermore, it is exciting to know that the authors have now obtained evidence of red-shifted absorptions, which further strengthen the exocomet scenario.

I only have a couple of minor comments. I would perhaps add a question mark at the end of the title since exocometary activity is not yet unambiguously confirmed.
And, at the end of the "Stationary sodium lines" section, perhaps the authors could quickly assess the origin of the -22 and -16 km/s (pseudo) stationary lines. This could be easily done by checking the archival spectra of other stars at a small angular separation and similar distance from Earth (if possible); if any of these lines are observed in other star this provides a strong indication of the component originating from the ISM. See for instance the paper by Iglesias et al. (2023) on the blue-shifted lines in eta Tel. It would not take too much work and this could provide a clearer picture on the origin of these two absorptions.

Given that the authors now have detected red-shifted sodium absorption in new UVES observations, I wonder if it wouldn't be preferable to hold the paper for a bit and re-submit once the UVES observations have been analysed so they could provide stronger evidence supporting the exocomet scenario. This is up to the authors of course, but I think this would be worth including in the paper as it strengthen the conclusions significantly.

Finally, congratulations on your work. I have no further comments, the paper has been thoroughly improved in my opinion.

(Remarks on code availability)

Reviewer #4

(Remarks to the Author)
The paper entitled "Sublimating exocomets in the PDS 70 system" by Novais et al. presents the spectroscopic detection of exocomets transiting the young protostar PDS 70.

The results presented in this manuscript are of major importance, as they put forward a plausible and physically consistent scenario for the origin of the water detected by JWST in this well-studied young planetary system. More broadly, given that only a small number of exocometary systems have been identified so far, PDS 70 represents a particularly valuable laboratory for the study of exocomets. With its high rate of exocomet transits, further supported by the authors' most recent UVES observations, this system is likely to become a prime target for future observations.

In the previous round of review, several important concerns were raised, in particular the possibility that the detected spectroscopic absorptions could instead originate from stellar winds. The authors have addressed these issues convincingly, notably through new UVES observational data showing redshifted absorption features, and a more cautious wording of the interpretative statements. These revisions substantially strengthen the manuscript and make the proposed exocometary interpretation more compelling.

In my view, the most important new element is the recent UVES observations showing redshifted absorptions, which provide additional support for the exocomet scenario. However, this key information is currently presented only in the response to the referees and is not made available to the readers of the paper. I therefore strongly encourage the authors to include this material, or at least a clear summary of it, in the manuscript.

Overall, the authors have satisfactorily addressed the comments raised on the previous version, and I have no major remaining concerns. In particular, the description of the fits to the absorption lines has been significantly improved, especially with Figures 2 and 7, and the new Tables 3 and 4 are welcome additions. I also appreciate the estimate of the total sodium mass converted into the typical size of an evaporated planetesimal, of about 1 km in diameter, which is consistent with the exocomet scenario.

The revised Figures 2 and 7 are useful additions, as they allow the reader to identify more clearly each detected feature and its measured properties. Tables 3 and 4 are also valuable, although they remain somewhat difficult to read in their current format. I suggest making them available in electronic form.

In short, I recommend publication of the manuscript after minor revisions.

In particular, I suggest

1) To include in the manuscript a description, or at least a clear summary, of the most recent UVES observations showing redshifted absorption features, since these provide important additional support for the exocomet scenario.

2) To make Tables 3 and 4 available in electronic form, as they contain useful information but are difficult to read in their current format.

3) To ensure that the wording throughout the manuscript remains appropriately cautious when discussing the exocomet interpretation.

(Remarks on code availability)
In contrast to the previous review round, I was unable to access the sodium-line fitting code used in this work, despite the authors' statement that it is publicly available through Code Ocean at https://codeocean.com.

Reviewer #5

(Remarks to the Author)
Dear authors,
Thank you for your careful revision and for thoroughly addressing the concerns raised in the previous referee report. The authors have responded to all points raised, and the improvements are clearly reflected in the manuscript. The clarified plots, the more detailed explanation of the observed blueshifted lines, and the restructuring and reordering of the sections have all contributed to making this a significantly stronger manuscript.

A few minor clarifications would further strengthen this work. The remaining points to clarify are outlined below.

I co-reviewed this manuscript with one of the reviewers who provided the listed reports. This is part of the Nature Communications initiative to facilitate training in peer review and to provide appropriate recognition for Early Career Researchers who co-review manuscripts.

Minor points linked to the authors' response to the referee reports:

Page 9
Section: "Exocomet as the origin of the variable sodium lines"

Paragraph 1: The minimum sodium mass estimates quoted here would benefit from a clearer justification of how these values are derived. A reference to the relevant section in the manuscript would help.

Paragraph 2: The discussion comparing PDS 70 and Beta Pic would benefit from some restructuring to improve clarity. As currently written, it is stated that Beta Pic ionises sodium more efficiently than PDS 70, yet sodium absorption features

appear to be clearer and more frequent in PDS 70. A clearer explanation of why this is the case would help the reader.

We suggest reorganising this section as follows. When introducing Beta Pic, the discussion primarily focuses on the CaII H&K lines. It would therefore be natural to first address the lack of detected variability in these lines for PDS 70. The discussion can then transition to the sodium absorption features detected around Beta Pic and how they compare to those reported in this work (e.g., differences in detection frequency and possible explanations for them). Together, these points can then be presented as a source of uncertainty regarding the exocometary origin of the signal.

Page 29
Section: “N-body simulations”

The multiple changes to the text and answer to the previous comments help to clarify most of the issues that were raised before. However, a few minor points should still be addressed:

- Text should say that stopping timescales are longer than the age of the system for planetesimals larger than “x” km. The absorption features could be caused by planetesimals/exocomets with a wide range of sizes, and so it is important to say for what planetesimal sizes the N-body simulations are valid.

- The new panels in Figure 4 further clarify the dynamical picture of how particles are evolving and transported to the inner system. However, there are still details that need to be clarified. Mainly, how are particles far from the chaotic zone of c excited into highly eccentric orbits before experiencing close encounters / scattering events with planet c? Panel c in Figure 4 shows that there are regions in the outer disk where particles can be excited, while this does not happen in other regions. For example, there is a “gap” between 70 and 80 au where no particles are scattered to the inner regions. With 3 planets, this changes, and no particles further than 75 are scattered inwards. Mean motion or secular resonances could be responsible for exciting those particles far from the inner edge of the outer disk, which could explain why there are gaps or islands in panels c and f. The authors could plot the eccentricity vs semi-major axis as a function of time in the outer disk, overplot the MMRs of c and even the secular resonances for the 2 planet case. Alternatively, the text could highlight these gaps/islands and list what processes may be exciting those particles further out. Note that the excitation will compete with gas drag, and so this process might preferentially excite only the largest planetesimals.

- The choice of recording particle parameters at the time they cross the gap, rather than at the end of the simulation, is acceptable provided appropriate caveats are given. The uncertainty introduced by the N-body simulations is acknowledged in lines 574-585, which is good. However, the “N-body simulation” section would benefit from a more explicit justification for this choice – specifically, clarifying that subsequent orbital evolution after “crossing the gap” is not the focus, and that the primary goal is simply to demonstrate that material transport from the outer to inner disc is possible.

- The choice of initial particle inclinations is well investigated and clearly justified in the authors’ rebuttal. It would nonetheless be worthwhile to include a brief justification in the manuscript itself for the adopted range of ±10deg. This could be as simple as a short statement acknowledging that similar conclusions are reached with lower initial inclinations.

(Remarks on code availability)

Reviewer #6

(Remarks to the Author)
I co-reviewed this manuscript with one of the reviewers who provided the listed reports. This is part of the Nature Communications initiative to facilitate training in peer review and to provide appropriate recognition for Early Career Researchers who co-review manuscripts.

(Remarks on code availability)

Version 2:

Reviewer comments:

Reviewer #3

(Remarks to the Author)
Dear authors and editors,

I am happy with the current version of the manuscript and recommend for publication.
I have no further comments. Congratulations on your work. Looking forward to see what the analysis of the full UVES dataset reveals.

Kind regards

(Remarks on code availability)

Reviewer #4

(Remarks to the Author)
The paper entitled "Sublimating exocomets in the PDS 70 system" by Novais et al. presents the spectroscopic detection of exocomets transiting the young protostar PDS 70.

The authors have now addressed all the major and minor comments raised by the reviewers. The manuscript presents a detailed and convincing case in support of the exocomet scenario. In particular, the inclusion of UVES spectra in the new Figure 6, as suggested by Reviewers 3 and 4, is a valuable addition.

I recommend publication of the manuscript in Nature without further revisions.

(Remarks on code availability)
With the new link provided by the authors, I am now able to access the codes.
I verified that the code is publicly available and that all required files appear to be included.
However, I did not attempt to run it or to assess its proper functioning.

Reviewer #5

(Remarks to the Author)
Dear authors,
Thank you for your careful revision and for thoroughly addressing the concerns raised in the previous referee report, particularly those regarding more explicit caveats. The paper has improved significantly and now satisfies all our previous concerns.

We noticed one remaining typo in a newly added section (page 30, line 1358: missing "[in] the inner regions…"). Once this is corrected, the paper will be ready for publication.

Congratulations on a very nice piece of work, which will generate great excitement in the community.

I co-reviewed this manuscript with one of the reviewers who provided the listed reports. This is part of the Nature Communications initiative to facilitate training in peer review and to provide appropriate recognition for Early Career Researchers who co-review manuscripts.

(Remarks on code availability)
NA

Reviewer #6

(Remarks to the Author)
I co-reviewed this manuscript with one of the reviewers who provided the listed reports. This is part of the Nature Communications initiative to facilitate training in peer review and to provide appropriate recognition for Early Career Researchers who co-review manuscripts.

(Remarks on code availability)

Open Access This Peer Review File is licensed under a Creative Commons Attribution 4.0 International License, which permits use, sharing, adaptation, distribution and reproduction in any medium or format, as long as you give appropriate credit to the original author(s) and the source, provide a link to the Creative Commons license, and indicate if changes were made.
In cases where reviewers are anonymous, credit should be given to 'Anonymous Referee' and the source.
The images or other third party material in this Peer Review File are included in the article's Creative Commons license, unless indicated otherwise in a credit line to the material. If material is not included in the article's Creative Commons license and your intended use is not permitted by statutory regulation or exceeds the permitted use, you will need to obtain permission directly from the copyright holder.
To view a copy of this license, visit https://creativecommons.org/licenses/by/4.0/

NCOMMS-25-98380-A
“Sublimating exocomets in the PDS 70 system”

Dear editor, dear referees,

We thank you for your very thorough review of our manuscript, and for the various helpful questions and answers. Below, we provide reactions to each of the comments that you have raised. In summary, we have taken to heart your (and also previous) referee’s concerns that, in principle, the exocomet hypothesis is difficult to distinguish from variable disc winds, mostly due to the fact that these exocomets seem to be only blue-shifted.

Our most important and impactful change to the text, is that we have now rebalanced the narrative, putting the hypothesis that additional absorption lines in sodium could plausibly be considered to be due to disc winds, at least at first sight, both in the abstract and in the main text. We then continue to lay out our main challenges to the disc-wind model, which are now in order of importance:

– The strong day-to-day variability in number of components and radial velocity, which appears to be atypical of disc wind lines (even if these are sometimes slightly variable too).
– The limited fill fractions, implying clumpiness that is typical of exocomets (ruling out diffuse disc winds).
– A model estimate of the expected mass loss rate due to disc winds, which implies that the column density expected for sodium disc winds is around 2 orders of magnitude higher than expected for this system (rightly pointed out by Referee #4 as being 2 orders of magnitude where we initially claimed 1 order of magnitude) — yet we now clearly acknowledge that this is a model that is based on significantly uncertain parameters, and we treat this model as supportive evidence only.

In our replies, we have also provided important additional information:
– We have been obtaining follow-up observations with the UVES spectrograph, that at least on two occasions seem to show red-shifted events.
– We carried out more tests using our N-body simulator, showing that a significant number of particles can reach high inclinations and high eccentricities, and that this does not seem to depend strongly on our initial conditions (in particular the spread of initial inclinations).
– We looked more closely at the Ca II H&K lines, and show that the flux of the system is so low that the stellar continuum is not confidently detected (also in the case of our new UVES data), precluding any detections of exocomets in Ca II at this time.

Altogether, we now frame the exocomet hypothesis as a plausible alternative to the interpretation that these observations are due to an unusual form of disc winds, and we end the main text of the paper by providing a number of observations that could help confirm the exocomet hypothesis further.

We have rewritten large sections of the main text, and also reorganised essentially all sections, and therefore the main text is not highlighted to show comparison to the previous version, because these texts only overlap sporadically.

Sincerely,

Aline Novais (corresponding author), H. Jens Hoeijmakers, Alexandra Stockwell Murphy, Bibiana Prinoth, Anders Johansen, Klaudia Jaworska, and Luan Ghezzi (contributing authors)

Responses to Comments from NEW Reviewer #3

Reviewer #3 (Remarks to the Author): The authors have performed a detailed examination of archival HARPS observations of the system PDS 70 and have found variable absorption features they believe are attributable to exocomets in highly elliptical orbits around the star. The presence of exocomets in this system would be of high impact, as no exocomets have been detected before in a K-type star via spectroscopy. In addition, the system is particularly young and hosts two protoplanets. This would advance our understanding on planetary systems formation and evolution.

The authors have done a good job in addressing several aspects to assess the exocomets scenario including comparison with stellar winds, accretion, stellar spots, variability in other T Tauri stars, and simulations of orbital inclinations and efficiency of scattering of exocomets.

However, given the evidence presented in this paper, I do not think these strongly confirm the exocomet scenario. I agree with the last comment of reviewer #1 and also think the results should be significantly toned down. Instead, I'd say that exocomets might be "a possible explanation" or that "the exocomet scenario cannot be ruled out". I do not see this study as conclusive evidence for exocomets and I explain why:

Reviewer #3: The N-body simulations performed by the authors show that an average of 0.47% of planetesimals are observed to have crossed the gap towards the inner disc (with confirmed protoplanets b and c, and higher percentage if potential planet d was considered). Then, about 10% of these planetesimals would evolve into high orbital inclinations, enough to be transiting the line of sight (or 6% if considering planet d). Yet, the authors found 42 blue-shifted Na I absorption line components which they attribute to exocomets within only 18 epochs of observations. It seems unlikely to be able to detect such high level of exocometary activity (comparable to Beta Pictoris) when the odds of exocomets transiting are quite low. Beta Pic has a particularly favourable orientation (almost exactly edge-on), whereas PDS 70, unfortunately, does not.

AN as Authors: We agree with the referee that as it stands, the absolute numbers of planetesimals that are scattered into the line-of-sight may be too small to explain the frequency of events observed in the 2018 spectra, due to the unfavourable inclination of the system (compared e.g. to Beta Pictoris). However, there are two important facts about our simulation that we wish to underscore:

– The orbital evolution of planets scattered into the inner system is highly dependent on the presence of any additional planets there. At this time, we have no way of knowing whether such planets exist, and even the properties of the known planets (in particular their masses) are observationally poorly constrained. It may turn out that our exocomet hypothesis will depend on the existence of additional (perhaps inclined) planets in the inner system, and we expect that this will become testable when ELT-era direct imaging observations become possible. We have added this to the conclusion (see lines 553–586).

– In the Beta Pictoris system, it is known that exocomets occur in "families", with a common orbital evolution (or even common parent bodies) that transit at different times. It may therefore be that a small number of planetesimals initially scattered inwards may create a large number of events. We actually have marginal evidence that this could be happening, considering the fact that the 2018 data is very rich in exocomet transits, whereas the 2020 data is largely quiet (see Section "Blue-shifted exocomet lines").

We therefore wish to emphasise that the fraction of planetesimals that evolve onto high inclinations and ultimately perhaps transit cannot be compared with the statistics of transit events that are apparently in the HARPS data. Even in the case of Beta Pictoris where extremely good exocomet

statistics are available, N-body simulations are now only able to explain the broad properties of the distributions, but not their absolute frequencies (see e.g. Beust et al. 2024 and our recent work Jaworska & Hoeijmakers 2026). All in all, the purpose of our simulation is to show that it is plausible that planetesimals cross the gap from the outer (> 54 AU) region inwards, and that even without additional planets in the inner system, eccentric and inclined orbits can, in principle, be attained.

Reviewer #3: Regarding the disc winds, these estimations are model dependant and based on several assumptions. Even the mass accretion rate values for T Tauris can have uncertainties of nearly an order of magnitude (see some of the Manara's papers on accretion rates). So I think the column densities being about an order of magnitude higher than that expected for disc winds is not a strong argument in favour of the exocomet scenario as these values can be within uncertainties and thus compatible with winds.

AN as Authors: We thank the referee for their expertise, and we agree with the referee's statement that our assessment of the mass loss in disc winds is weak in the presence of order-of-magnitude-level uncertainties. This has now been properly dealt with in the text (see Section "Disc winds?"). We do agree with the overall challenge that the lines we propose to be exocomet lines are in principle difficult to distinguish from the disc wind phenomenon (see also our response to several other comments below). We have therefore chosen to rebalance our narrative to, early on in the text, explore and give credence to the disc wind-explanation for these lines.

Regardless of the value that is (or isn't) in the estimation of the disc wind mass loss based on the stellar accretion rate, our main argument for introducing the exocomet hypothesis as an alternative is based on the fact that there is short time-variability, in both the number of absorbing components and their radial velocity, combined with their sometimes significantly partial fill fractions, as being highly anomalous for typical disc winds of T Tauri stars, especially of the later age where the accretion rate is relatively small.

In the new version of our text, we now first discuss disc winds, before moving on to how these variabilities might be better explained by transiting sublimating material.

Reviewer #3: In the section about the origin of the blue-shifted absorption lines, the authors say that the detections are biased towards the blue-shifted side of the sodium lines because the red-shifted part of the spectrum is strongly contaminated by the stellar accretion emission line. I disagree with this statement. When I look at figure 1b, I see that the accretion line is well centered at the stellar radial velocity, so there should not be any bias towards blue-shifted or red-shifted detections. Although it is true that blue-shifted absorptions are plausible for exocomets transits, the lack of red-shifted absorptions makes me more skeptical about the exocomet scenario, as the presence of only-blueshifted absorptions is consistent with winds.

AN as Authors: We agree with the referee that our earlier explanation of the red-shifted features being unobservable is indeed incomplete. At red-shifted velocities away from the accretion line, we should expect to see events at higher velocities if red and blue shifted transits were equally likely. We see highly blue-shifted events, yet we do not see highly red-shifted events, and therefore the preference for blue-shifts is not just an observational bias.

We have two hypotheses for explaining a preference for blueshifts:

– The first follows the scenario of Beta Pictoris, where a preferential red-shift is caused by the population of exocomets being in a particular family of orbits, shaped by mean-motion-resonance with

Beta Pictoris c. It comes down to the chance orbital alignment (which in Beta Pictoris is time-dependent due to precession with a ~10000 year periodicity) whether the orbits result in red-shifts or blue-shifts during transit.

– The second is that it is currently only an assumption that the observed radial velocity actually corresponds to the orbital motion of the sublimating nucleus. Depending on the environment (notably gas density and ionisation fraction), the sodium in the tails is blown away from the star, and reaches significant tailward velocities due to radiation pressure and/or solar wind effects. This is unlikely to explain the highest velocity events (many 10's of km/s blue-shift) but may significantly contribute to the observed asymmetry between red and blue-shifts. Sodium atoms in the tail of Mercury have a velocity of up to 11 km/s (Potter et al. 2010).

Over the course of this year, we have started to acquire new UVES spectra to follow up this phenomenon. In at least two occasions, we observe signs of at least one red-shifted sodium line component (2026-01-18 and 2026-03-22), as well as low-velocity (~10 km/s) and high-velocity blue-shifted lines (see additional figure below). These observations are still being obtained so our analyses are not yet final, but we take this as evidence that these transient absorption features are not exclusively blue-shifted.

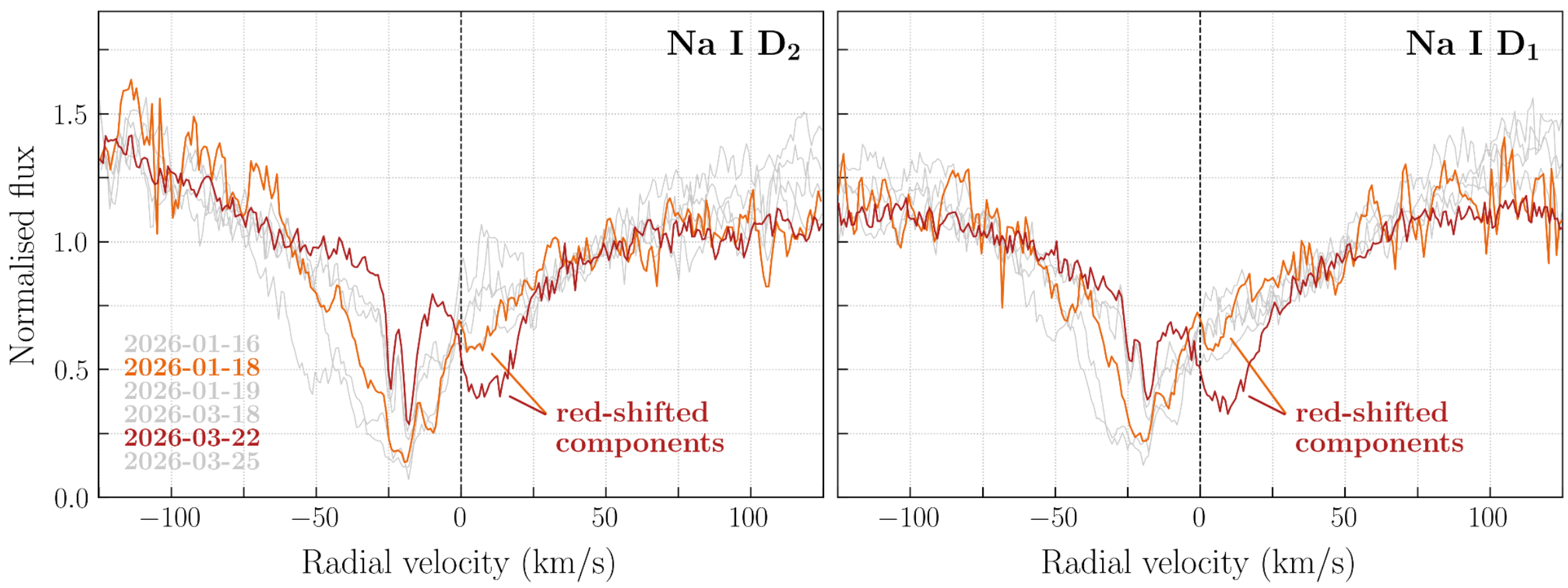

Additional Fig.: UVES spectra acquired in January and March 2026 (Program ID 0116.C-0329, PI A. Novais).

Reviewer #3: Also the lack of CaII absorption makes the exocomet scenario less convincing. As the authors mention in their analysis; absorptions are expected in the CaII H&K lines (if produced by exocomets sublimation). In order to prove that the lack of absorption in the UV lines is due to bad SNR, I'd suggest to inject in the spectra the expected signal of an absorption in the CaII K line. Since for exocomets, column densities for CaII are expected to be higher than for NaI (Kiefer et al. 2014), perhaps it would be a good idea to adopt a detection limit for similar total column densities: N(CaII K+H) >= N(NaI D1+D2) and similar optical depths to NaI, and compare that to the deepest NaI detection to check the detectability in CaII K.

AN as Authors: Below we plot the Ca II K line as observed by HARPS, to make clear our point about SNR. The flux at these wavelengths is so low that the stellar continuum is essentially not detected. All that is detected is the variable Ca II emission line core at 0 km/s, and its variability. We have now made this more explicit in the text (see Section “Exocomets as the origin of the variable sodium lines”).

Unfortunately, our new UVES observations (see above) also do not obtain sufficient flux at these wavelengths to observe the stellar continuum.

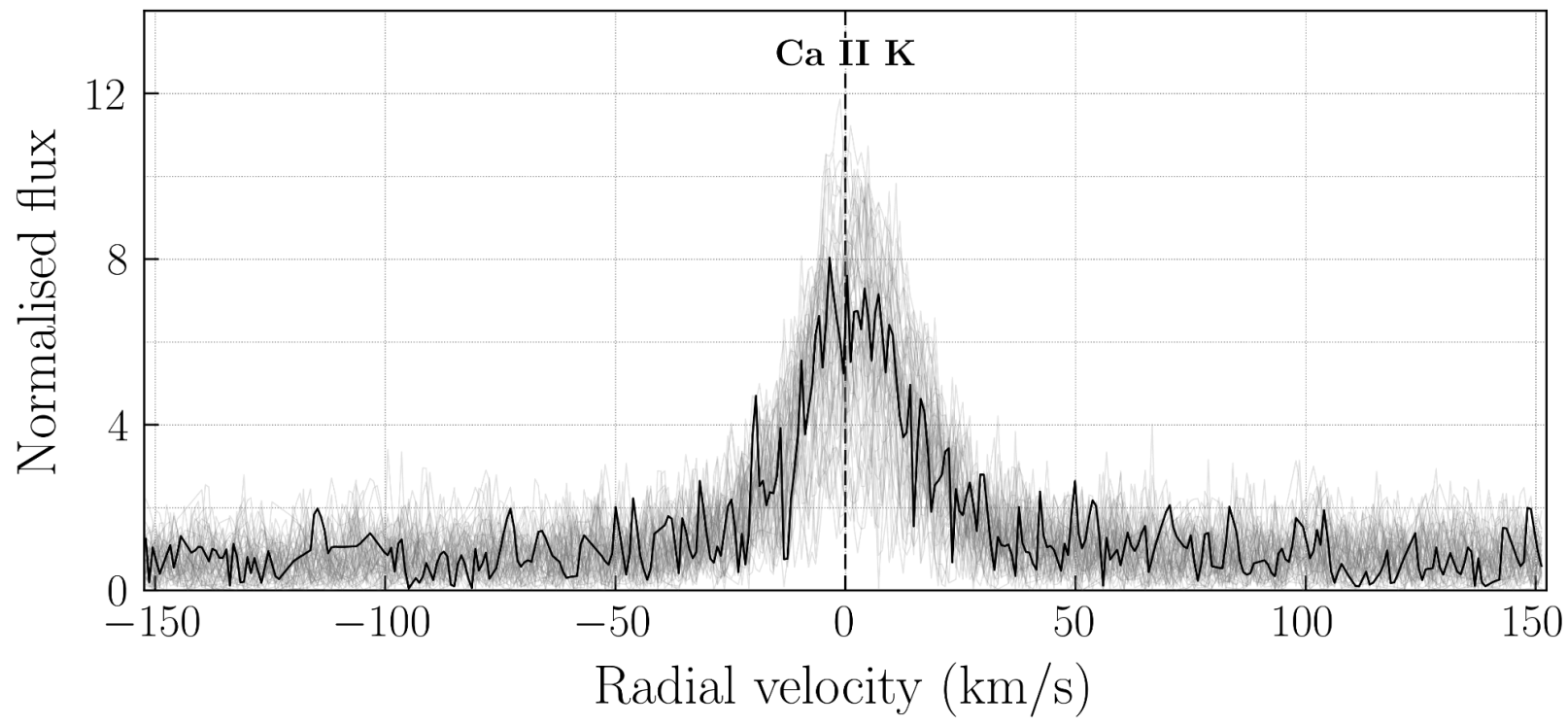

Additional Fig.: HARPS data around the Ca II K line. The first epoch is highlighted in black.

Reviewer #3: A side concern; the authors' response to reviewer 1 comment regarding the ISM contribution. I believe a significant part of their response is very wrong. The authors claim it is "highly unlikely" for the ISM to create the pseudo-stable absorption feature at -19 km/s and do NOT believe that the observed lines at -19km/s can plausibly be attributed to the ISM. The authors should be aware that the study of Redfield & Linsky (2008) is limited to a distance of 15pc. Any interstellar cloud beyond this distance will not appear in this survey. The LIC is only the Local Insterstellar Cloud, which is the cloud where the Solar System is embedded. The interstellar medium is filled with clouds with a wide range of densities. Since PDS 70 is at a distance of ~112pc (according to Gaia EDR3), then it is very likely that there are more interstellar clouds (beyond the LIC) crossing the line of sight of the star. The more distant the star, the more ISM material can be crossing its line of sight. Examples of ISM absorptions with NaI column densities in the order of 10^12 can be found in Welsh et al. (2010); please look at figures 2 to 5. In some of these examples the line depths exceed 50%. Therefore, I think it is highly plausible that some of the pseudo-stable blue-shifted absorptions found in the spectra are due to ISM contribution. Of course, these do not explain the variable absorption features or any correlation with stellar rotation, but it would be a good idea to assess any possible ISM contribution in the spectra to better account for additional absorptions.

AN as Authors: We thank the referee for rectifying our previous response, and we now fully agree. At the same time, the referee acknowledges that line variability should not be expected to be caused by the ISM, and unfortunately, the case may well be that the ISM line is overlapping with a variable (in amplitude) disc wind line.

We have rewritten the text considering the possibility of a ISM contribution (see Section "Stationary sodium lines").

Reviewer #3: Overall, I think given that the system does not have a favourable orientation, and the lack of CaII detection, and the system being a T Tauri with ongoing accretion and likely to have winds, and only blue-shifted absorptions; exocomets do not seem as the most likely explanation for the variable absorption features detected in NaI. Winds, in my opinion, seem like a more plausible cause. I hope the authors can prove me wrong in the future, as this would be certainly a very interesting discovery, but the evidence presented so far is not strongly convincing. Hopefully, the authors could obtain stronger evidence in future observations such as an asymmetric transit in photometry or simultaneous CaII and NaI detections in spectra with better SNR. I'd suggest trying to obtain UVES or ESPRESSO observations as these could achieve a better SNR than HARPS.

AN as Authors: As we have stated in previous comments, we agree with the general picture that disc winds, in principle, form a likely challenge to our hypothesis. As explained in the previous comments, we have now rebalanced the text to discuss winds up-front, and presenting the exocomet hypothesis as an alternative scenario, motivated by the fact that observationally, the strong variability seen in these lines is anomalous from the point of view of disc winds (see above).

Responses to Comments from NEW Reviewer #4

Reviewer #4 (Remarks to the Author): The paper entitled "Sublimating exocomets in the PDS 70 system" by Novais et al. presents the spectroscopic detection of exocomets transiting the young protostar PDS 70.

The results presented in this manuscript are of prime importance, as they propose a plausible and physically consistent scenario for the origin of the water detected by JWST in this well-studied young planetary system. In a broader context where only a handful of exocometary systems have been identified to date, this system represents a particularly valuable new laboratory for the study of exocomets. It is therefore likely to become a prime target for future observations with the most advanced astronomical facilities.

The two previous referees raised several important concerns, notably regarding the inclination of the exocometary orbits, the possibility that the detected spectroscopic absorptions could originate from stellar winds (Referee #1), and the non-detection of Ca II (Referee #2). The authors have now convincingly addressed all these points through the inclusion of new numerical simulations, a detailed quantitative analysis of the stellar wind scenario, and an improved assessment of the signal-to-noise ratio demonstrating that the Ca II non-detection is fully consistent with observational limitations. These additions significantly strengthen the manuscript and make the proposed exocometary interpretation compelling.

I have a few minor comments detailed below :

Reviewer #4: 1) Most important comment :
Several tables listing the adopted priors are provided (Table 3 and subsequent tables), but I could not find any table summarizing the numerical results of the fits to the 42 individual absorption signatures. Figure 3 alone is insufficient for this purpose. A comprehensive table reporting the best-fit parameters (and associated uncertainties) for each absorption event is currently missing in the paper.

AN as Authors: We agree with the referee's comment that it would be useful to have access to this additional data. We have updated the table with priors and included tables with the best-fit values for each absorption line, for each epoch (see Tables 2–4).

Reviewer #4: 2) In the Introduction, when discussing photometric detections of exocomets, it would be appropriate to also cite the recent studies by Pavlenko et al. (2022), Lecavelier Des Etangs et al. (2022), and Kiefer et al. (2023).

AN as Authors: These citations are now included (see Section "Exocomets as the origin of the variable sodium lines").

Reviewer #4: 3) Line 122.
A citation to the reference [20] in addition to the reference [12] would be useful, as it provides tools to quantify the covering factor (as used in the present work).

AN as Authors: The text was rearranged, but the citation was also included (see Section "Exocomets as the origin of the variable sodium lines").

Reviewer #4: 4) Line 183.

Converting the inferred sodium mass into a characteristic size of the parent body would be helpful for the reader, for instance by assuming a meteoritic sodium abundance. This would allow a direct assessment of whether the inferred masses are realistic. A rough estimate suggests that a kilometer-sized body could already account for a few 10^9 kg of sodium, but this deserves to be quantified more carefully.

AN as Authors: We agree with the referee that this is a useful extension. We indeed find that entirely vaporising kilometer-sized bodies can account for these minimum-masses, and we have now added a few sentences on this (see Section "Exocomets as the origin of the variable sodium lines").

Reviewer #4: 5) Line 185.
The comparison between the measured sodium column density and that reported for comet McNaught (C/2006 P1) appears somewhat hazardous. In the latter case, the sodium measurement was obtained in emission and probes a limited region along the line of sight, making a direct comparison with the present absorption measurements potentially misleading.

AN as Authors: We agree with the referee that the measurement principle in these work on comet McNaught and Hale-Bopp are different, as these papers attempt to estimate the sodium column in a limited part of the tail. We have removed this comparison from the text, as we agree that the above estimates (with km-sized bodies being needed to create our observed cloud masses) already show that the cloud masses we obtain are within the realm of what is seen in bodies in the solar system. We have included this in the text (see Section "Exocomets as the origin of the variable sodium lines").

Reviewer #4: 6) Line 257.
It seems that the sodium column densities measured in the absorption lines are two, rather than one, orders of magnitude higher than those expected from the stellar wind scenario.

AN as Authors: We thank the referee for the rectification. To be clear, our numbers for the exocomets are now (after repeating the fits) ranging from 2.4e11 to 7.1e12 cm^-2, while the estimates from the mass-loss model range from 4.4e9 to 6.6e10 cm^-2. This is almost (but not exactly) two orders of magnitude, and we kept these statements deliberately conservative, calling it one order of magnitude. Where relevant, we have now rephrased this to "at least nearly two orders of magnitude". By the face of it, we see this comparison as somewhat convincing, but we do agree with some comments by the other referees that the mass loss model can be seen as insecure, due to the considerable uncertainties on the various parameters. We therefore treat this comparison as supporting evidence, while we consider the variability and partial fill fractions to be our main lines of evidence (see our replies to Referee #3, and also Section "Disc winds?").

Reviewer #4: 7) Line 905.
It is unclear why the R line ratio is treated as a free parameter in Eq. 1, instead of being fixed to its expected value of 2. A justification for this choice, or a discussion of its impact on the results, would be welcome.

AN as Authors: We thank the referee for spotting this important detail. In the text, we had incorrectly mixed up the concepts of observed line ratio versus intrinsic line ratio (ratio of oscillator strengths). This has now been corrected in the text (see Section "Fitting Sodium doublet lines").

More importantly, we had indeed kept the ratio free when fitting the data, and the referee correctly points out that this is not scientifically justified nor necessary, while adding unnecessary complexity

and freedom. In our repeating of the fits, we have now fixed this parameter to 2, resulting in tighter posterior distributions, and in almost all cases, smaller inferred fill fractions. However, in most cases, the fitting results remain equivalent (because the fit preferred the correct line ratio of 2.0). And, importantly, our conclusions based on these results, are unchanged, and are now much more confident if anything.

Reviewer #4: 8) Line 1322. Caption of Fig. 3
The observed line broadening likely has a dynamical origin for exocomets located close to their host star. Here, the gas temperature may be largely independent of the distance to the star (see Vrignaud et al. 2025).

AN as Authors: We agree with the referee, and we have removed this statement from the text.

Reviewer #4 (Remarks on code availability): I verified that the code is publicly available and that all required files appear to be included.

However, I did not attempt to run it or to assess its proper functioning.

AN as Authors: We have updated the code with the new fits, which should be able to run normally.

Responses to Comments from NEW Reviewer #5

Reviewer #5 (Remarks to the Author): Dear authors,

Thank you for your careful revision and for tackling the concerns raised by the previous reviewers. A few parts of the manuscript still require clarification or a more explicit discussion. Below, the remaining points to clarify from previous referee reports are outlined, along with some additional comments.

I co-reviewed this manuscript with one of the reviewers who provided the listed reports. This is part of the Nature Communications initiative to facilitate training in peer review and to provide appropriate recognition for Early Career Researchers who co-review manuscripts.

Points linked to previous referee reports:

Reviewer #5: Page 9:
Line 394:
The number of planetesimals scattered to the inner regions would strongly depend on their initial parameters, such as how close they are to the chaotic zone of c, of resonances in the outer belt, their initial eccentricities and inclinations, as well as the presence or absence of gas that can damp the orbits of solids. The text could acknowledge that these fractions are highly dependent on the assumed initial conditions and neglect gas drag, and point the reader to the supplementary material. It would also be interesting, if feasible, to show how the rate changes when varying these parameters.

AN as Authors: We have expanded upon our description of the N-body simulations that we do, and have also carried out several tests that have relevance to several of the referee's comments, including this one. We refer the referee to our reply to comment "i) First, how many particles are scattered inwards…" below, where we address this issue of initial parameters and gas drag.

Reviewer #5: Line 410:
The quoted 10% rate may depend strongly on the initial inclination distribution given to the planetesimals, which is relatively high if planetesimals are born very close to the midplane, where most large grains are. The authors should either add a caveat here or run additional simulations to demonstrate that the result is robust to changes in the initial conditions.

AN as Authors: We refer the referee to our reply to comment "ii) I suggest that the authors examine…" below, where we address this.

Reviewer #5: Page 18:
I agree that any variable absorption features in the CaII H&K lines would be undetectable due to the low SNR of the spectrum at those wavelengths. The calcium doublet is predominantly used to investigate the level of stellar activity in late-type stars, and this activity manifests as a (sometimes variable) emission feature that dominates over the photospheric stellar absorption (e.g., Strassmeier et al. 1990; Bendahan-West et al. 2025). In the case of PDS 70, there is strong CaII H&K emission (visible in the PDS 70 spectra shown as an additional figure in the response to comments of Reviewer#2), which would hinder any possibility of observing variable absorption features due to exocomet transits. I suggest adding a note on this, as this would strengthen the justification for the lack of CaII H&K detections.

AN as Authors: We have properly included this in the text (see Section "Exocomets as the origin of the variable sodium lines"). Please also see our reply to Referee #3, where we provide a plot of the spectrum near Ca II K, showing how the stellar continuum is indeed essentially undetected.

Reviewer #5: Page 23:
"N-body simulations" section:
As mentioned earlier, the results of these simulations may depend strongly on the adopted initial conditions. There are three points that I find concerning:

i) First, how many particles are scattered inwards will strongly depend on how close those particles are to the outermost planet (PDS 70 c). If those particles are placed beyond ~3 Hill radii or the chaotic zone (e.g. Pearce et al. 2014, Marino et al. 2018), they may never be excited to an eccentric orbit to later be scattered by planet c. So, to make these results transparent, the authors could include a 5th and 6th panel in Figure 7 showing eccentricity vs semi-major axis for all particles at the end of their simulation, marking the minimum and maximum initial semi-major axes, and in addition, add the initial semi-major axis and eccentricities in colour of those that cross the gap (or as a density distribution as a function of semi-major axis as they start with zero eccentricity). This will determine whether the particles that cross the gap start all very close to the inner edge of the outer disc or not. If so, the authors should add a sentence stating that the inferred fraction strongly depends on the assumed initial semi-major axis of planetesimals.

AN as Authors: As suggested by the referee, we have included 2 new panels in Fig. 4 (previously Fig. 7) showing the semimajor axis vs. eccentricity of all the particles that crossed the gap, and tracing (with the straight lines) where they started in the outer disc. From these plots it is clear that although there is some preference for particles relatively close to planet c (54–60 AU) to be scattered onto high inclinations/eccentricities, although particles from further away also reach highly excited orbits, with periastron distances within 10 AU.

The histogram (panel a) makes the chaotic phase quite clear: In the first million years of the simulation, many particles (presumably close to the planet) get scattered inwards quickly, but after this, a steady flow of scattering is maintained for several million years.

In the case of three planets, planet c is assumed to have a much lower mass of 2.4 Mjup instead of 6.4 Mjup (Trevascus et al. 2025). In this case, the chaotic phase is not there (because of the much smaller Hill radii), and particles get scattered gradually for millions of years. Also, in this case, particles tend to arrive both from the inner region of the outer disc (54–60 AU) as well as from further away.

Putting all this together, we conclude that the inferred fraction of particles that crosses the gap is indeed dependent on the initial parameters, including the initial semimajor axes, but mainly on the assumed mass of planet c. It is true that the highest inclinations and eccentricities (when crossing the gap) are achieved by particles that started the simulation closer to planet c (54–75 AU), and this was included in the text (see Methods Section "N-body simulations").

Note that, as in the previous version, we have chosen to plot the particle parameters at the time they cross the gap, not at the end of the simulations. This is because we are interested in all particles that are ever able to cross the gap and approach the star, at any time over the course of 5 Myr. Subsequent orbital evolution; or the presence of additional planets in the inner system, would act to excite the orbital parameters further.

Reviewer #5: ii) I suggest that the authors examine how the results would vary if they assumed smaller initial inclinations (or even set them to zero) and justify using initial inclinations between ±10 deg. The initial inclination distribution should affect the calculated rates of planetesimals crossing the gap with sufficiently high inclinations, and these rates would likely be lower for smaller initial inclinations. This is because the higher the inclination, the more likely particles are to get a vertical kick by a planet that raises the inclination even higher. Since planetesimals may be born very close to the midplane, starting inclinations with ±10deg may significantly inflate the number of particles that cross the gap with 40 deg inclinations. The authors may not need to run additional simulations, and instead they could split their particles between those that start with low inclination (e.g. <1 deg) and those that start with high inclination (e.g >1 deg) to assess this.

AN as Authors: We have followed the referee's advice to verify the effect of the choice of initial inclination, by comparing between particles that were initialised with inclinations between ±5° and between ±1°. This comparison shows that particles migrate and obtain inclinations above the inclination of the disc at the time they cross the gap towards the inner disc, at roughly equal relative numbers regardless of the inclination distribution chosen. This is summarised in the table below, where we show the distribution of inclinations when crossing the gap, the fraction of particles that cross the gap and get inclined above 38.3°. Although we agree with the referee that in principle, the 10° spread in inclination that we have chosen may be large if you assume planetesimals to start in the midplane, we conclude from this test that the initial inclination is not a major factor. This is likely because the planets have small mutual inclinations themselves (0.8° in the two-plane case, and a large mutual inclination for planet d in the 3-planet case, see Trevascus et al. 2025), so the particles feel sufficient vertical kicks in both scenarios despite the fact that these two cases are quite dissimilar.

	**2 planets**	**2 planets**	**3 planets**	**3 planets**
Initial inclinations between	Fraction that cross the gap	Fraction with inclinations ≥ 38.3° when crossing the gap	Fraction that cross the gap	Fraction with inclinations ≥ 38.3° when crossing the gap
±10°	0.47% (out of 9800)	10.05%	2.90% (out of 8400)	5.76%
±5°	0.58% (out of 4845)	10.12%	3.15% (out of 4159)	4.47%
±1°	0.65% (out of 930)	12.72%	4.10% (out of 854)	3.60%

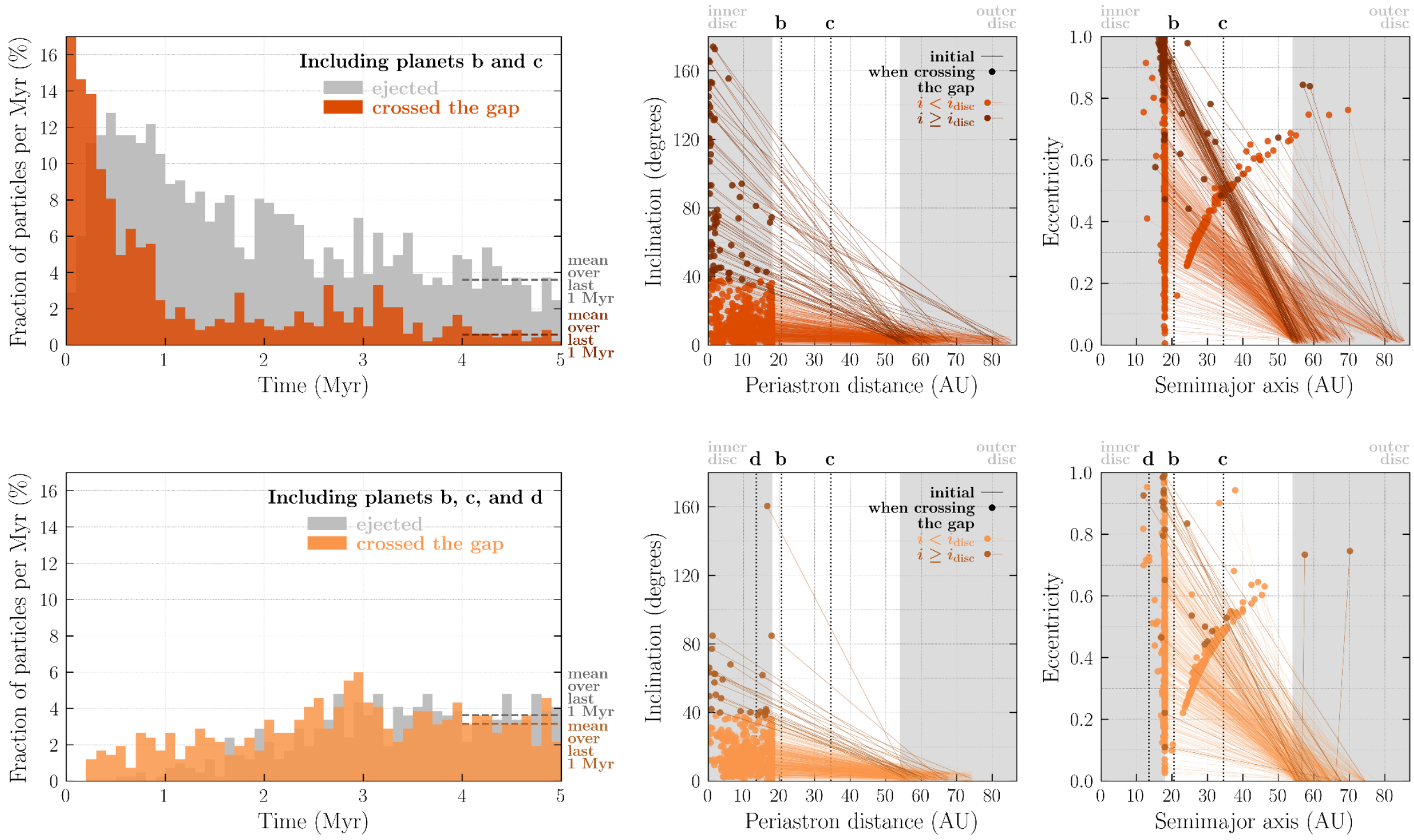

Additional Fig.: Same as updated Fig. 4 of the manuscript but only for the particles that were initialised with inclinations between ±5°.

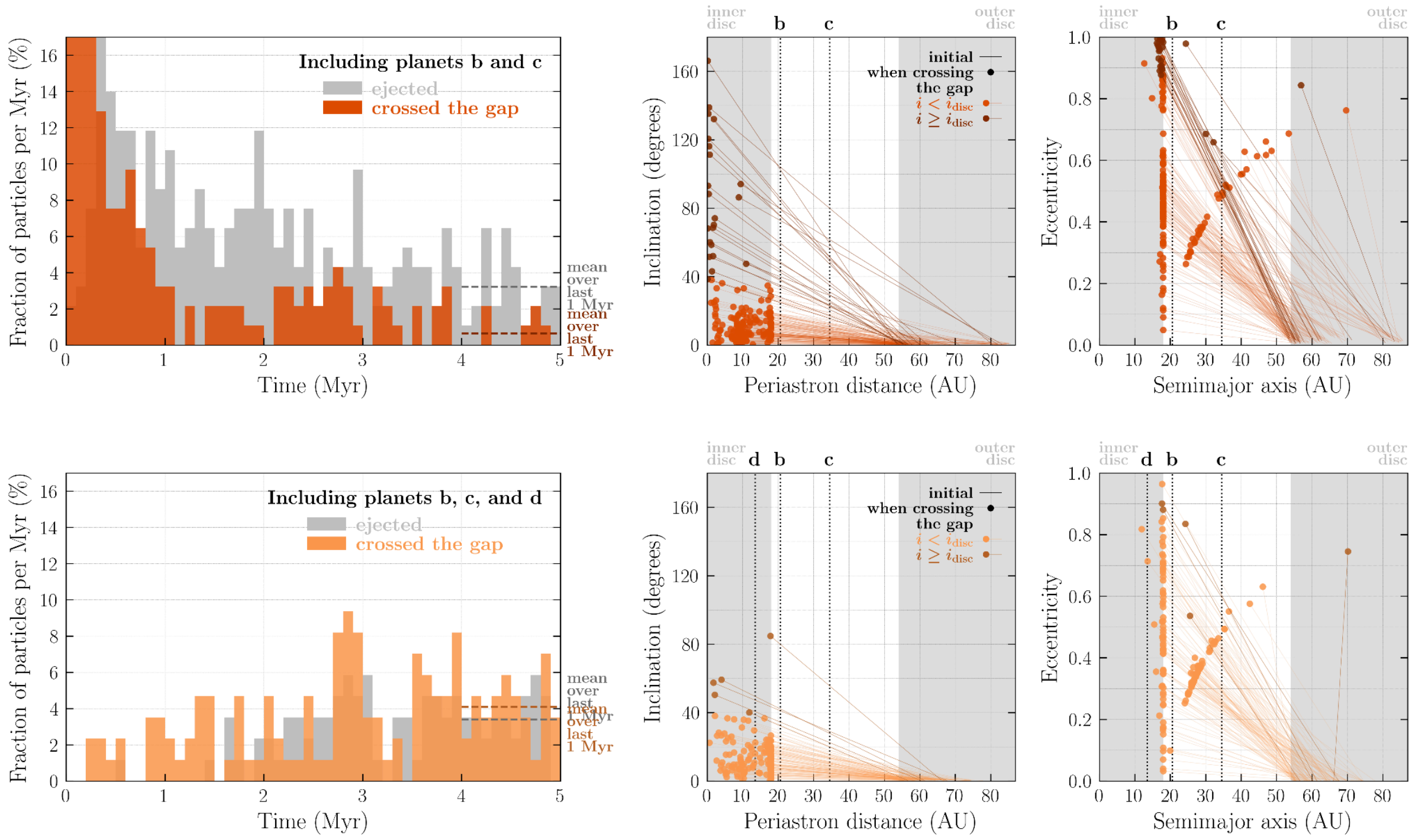

Additional Fig.: Same as updated Fig. 4 of the manuscript but only for the particles that were initialised with inclinations between ±1°.

Reviewer #5: iii) In addition, the simulations do not include any gas, which would damp planetesimal inclinations and thus could modify the inferred rates (e.g. Raymond et al, 2017). This damping may favour inward scattering relative to ejection, but it may disfavour the excitement of inclinations above 40 deg. I ask the authors to include this caveat in the text.

AN as Authors: We acknowledge the referee's statement. We are currently investigating the effects of gas by carrying out new simulations including a gas drag (deceleration) force. This work has been ongoing by a master student in our group since January 2025, and we can share some of the student's results that are relevant for the concern that the referee raises.

We estimated the stopping time (Eriksson et al. 2021, see additional figure below) of planetesimals, assuming a gas surface density profile (Portilla-Revelo et al. 2023), including the gap region where we assume the surface density to be depleted by two orders of magnitude (Keppler et al. 2019, Facchini et al. 2021, Portilla-Revelo et al. 2023). The stopping time is plotted in the figure below. The present-day gas density leads to a stopping time over 10 Myr for orbital distances greater than 10 AU. We therefore conclude that our gas-less, pure N-body simulation of planetesimals in the outer disc is unlikely to be affected by gas, at least until the planetesimals reach the inner system within 18 AU, and so we consider this result to be robust against the gas being neglected. The student is currently working to ascertain whether gas drag has meaningful effects on the orbital evolution interior to 10 AU, but this is not relevant for the N-body simulation that we have currently included in this study.

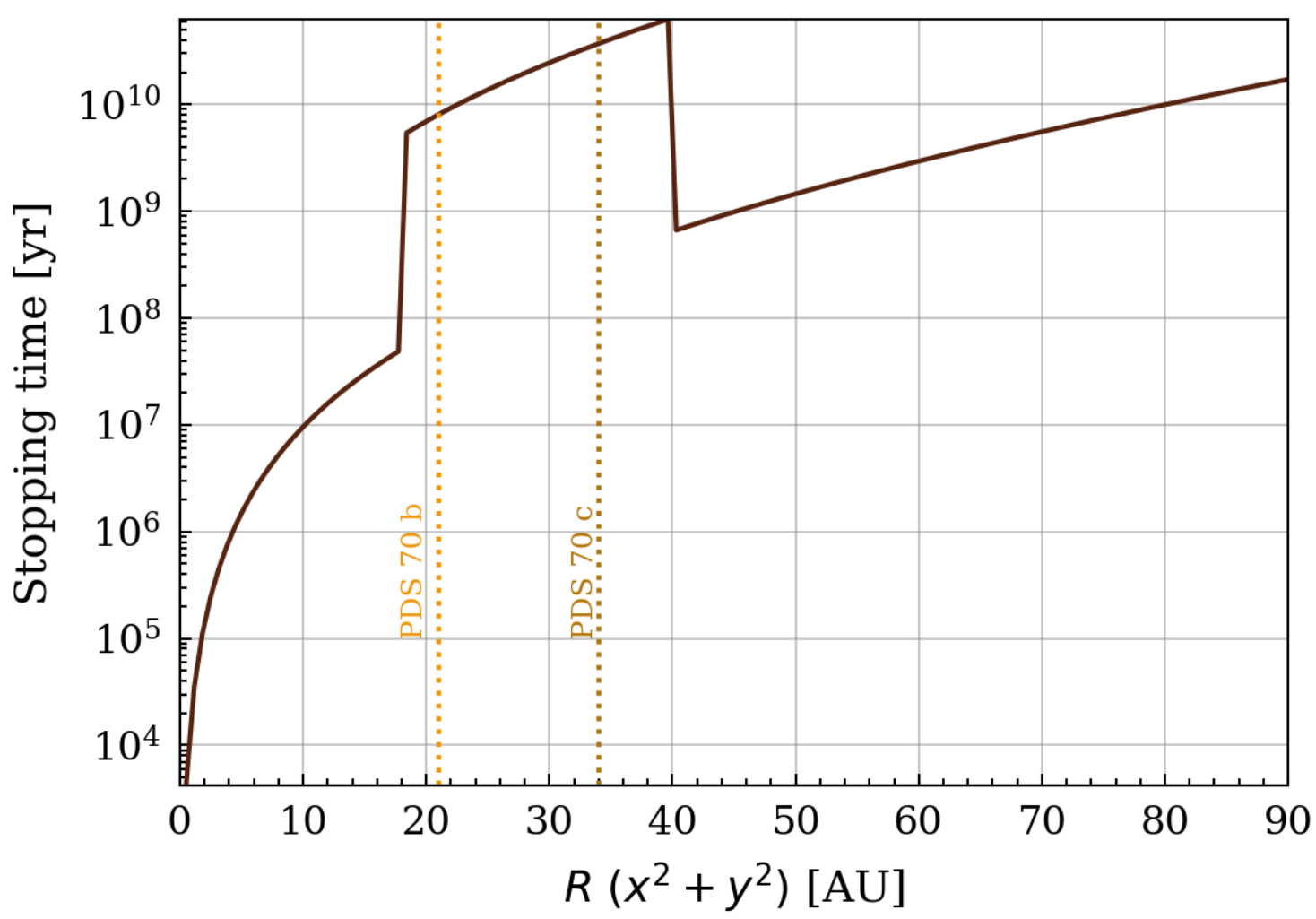

Additional Fig.: Stopping time of a planetesimal body as a function of radial distance to the star, using equation 11 of Eriksson et al. (2021, A&A 648, A112), assuming a planetesimal solid density of 1 g/cm^3 and a radius of 100 km. Stopping time is defined as the time where a particle is affected by the gas, suffering a change in velocity.

Reviewer #5: Page 27:

Fig 1b:

The "disc lines" would benefit from a more explicit discussion in the paper, particularly regarding their variability with the stellar rotation period and the possible ISM contribution (as explained in the responses to comments from Reviewer#1). Even if the feature is not attributed to the ISM alone, the authors should still discuss the potential ISM contribution in the text.

AN as Authors: We agree with the referee's comment. Referee #4 has also asked for more information regarding the disc lines, including fitting results. In response to that comment, we have now added tables of fitting results for all lines (see Tables 3 and 4), and also added a discussion about the variability in the methods section (see Section "Stationary sodium lines"). We also included more discussion about the ISM contribution. As stated in our reply to Referee #3, it is likely that the ISM contribution overlaps with one of the disc lines. This is now discussed in the text (see Section "Stationary sodium lines").

Reviewer #5: Additional comments:

Page 2:
Line 77: The authors could quantify "very short timescales" to indicate the typical variability timescale associated with exocomet transits.

AN as Authors: This has been modified in the text, where we quantify this as day-to-day variations throughout the paper (see Section "Exocomets as the origin of the variable sodium lines"). In principle, we expect exocomet transits to last anywhere between a few hours to ~1 day (Zieba et al. 2019).

Reviewer #5: Page 4:
Line 148-149:
The discussion of the surface fill fraction needs a caveat. The calculation appears to assume that the entire gas cloud is projected on the stellar disc. However, it is possible that the cloud only grazes the stellar disc. Because the surface fill fraction is a key parameter used to support an exocometary origin, this caveat and assumption should be stated, as it may affect some of the results and conclusions.

AN as Authors: With large clouds (significant fraction of the stellar radius) it is indeed likely that at any time, part of the cloud is not covering the star. However, such material would not be visible in the spectrum (as it is not in the line of sight), and therefore would not affect our fit of the data. Our formalism only assumes relative surface areas (not even spherical or circular shapes). The presence of cloud material out-of-transit only affects our interpretation of the mass of the transiting cloud, as part of the mass might indeed be missing from the line of sight. We therefore present our inferred masses as minimum masses.

We do agree with the referee that in lines 148–149, this was formulated incorrectly where we explicitly state that the clouds must be smaller than the star, which is indeed not true. Nevertheless, partial coverage does still imply that the cloud is spatially confined (even if it is larger than the star and grazing, this situation would be very unlikely if the cloud was much larger than the star), and therefore clumpy, which is at odds with diffuse disc winds. We have adjusted the text to remove this error (see Section "Fitting sodium doublet lines").

Reviewer #5: Line 179-180:
It is not clear why the analysis assumes optically thin lines, given that the optical depths are known and many of the lines are optically thick. On this line, "some" could be replaced by "many" to reflect the fact that a substantial fraction of the lines are optically thick (based on Fig. 3b).

AN as Authors: We agree with the referee's comment. The qualification that the analysis relies on optically thin lines was wrong, and this has been corrected (see Section "Calculation of sodium cloud mass"). We use the actual fitted optical depths, whether they are low or high.

Reviewer #5: Line 272:
Should A = f*pi*Rstar^2 be used instead of f*pi*(2*Rstar)^2? This change would lower the inferred mass loss rates and bring them closer to estimates from disc winds.

AN as Authors: Although 2Rstar is an assumption (and one could assume Rstar instead), we prefer to keep the 2Rstar factor in order to follow the same assumption used by Calvet (1997), also followed by Thanathibodee et al. (2020) and other studies, effectively implying that the wind is launched from an area around the star, with a larger projected size. We do agree that this choice affects the implications we might derive from the mass loss model. In our response to Referee #3, we have detailed that we have restructured the text to give more credence to the disc wind scenario up front, and the mass-loss rate calculation now plays a supporting role, as we agree that the magnitude of the uncertainties does not make the mass-loss rate estimation uniquely compelling by itself.

Reviewer #5: Page 8:
"Origin of the blue-shifted sodium lines" section:
The fact that all variable features are blue-shifted is interesting dynamically. It would be useful to include a comparison with the predominantly red-shifted features seen in the archetypal system Beta Pictoris, especially since much of the exocomet literature is built around that system.

AN as Authors: In the new version of the text, we have restructured the section on blue-shifted lines (see Section "Blue-shifted exocomet lines"), explaining that in Beta Pictoris, the preference for a red-shift is caused by a mean-motion resonance with the eccentric planet c in that system, leading to alignment of the orbital parameters (in particular the argument of periapsis, being forced into the same direction as the argument of periapsis of Beta Pictoris c). If such a mechanism would be acting in the PDS 70 system, this would imply the existence of additional planets much closer in than the planets currently known (Beta Pictoris c has a semi-major axis of about 3 AU). However, this is presently impossible to test.

In this section, we also include a discussion about other ways in which blue-shifts could be preferred. It is conceivable that the tail velocities of the sodium we are observing are significant. Often (in the case of Beta Pictoris), it has been assumed that the radial velocity of the exocomet cloud observed is the same as the Keplerian velocity of the sublimating nucleus, but this may not be true in all cases. In Beta Pictoris, there is significant braking of tail ions due to the high ionisation fraction of the medium, but in PDS 70, the neutral sodium may be more free to be blown in the anti-stellar direction, creating blueshifts.

We have some evidence that there are discrepancies between the Keplerian velocity and the observed cloud velocities: In some cases, we see a slight increase of the blue-shift (towards negative velocities), and this cannot be consistent with Keplerian motion: blue-shifted exocomets must, per definition, be decelerating as they recede from the star. We interpret such departures as possibly being caused by changing tail morphology during the transit, or a geometrical effect as the tail is rotating into our line of sight during the observations. Similar behavior is also sometimes observed in time-series observations of Beta Pictoris, see e.g. Hoeijmakers et al. (2025) where one clear event is observed.

These considerations are now included in the text (see Section "Blue-shifted exocomet lines").

Reviewer #5: In addition, line 370 suggests that the red-shifted part of the spectrum is strongly contaminated by the stellar accretion emission line, hindering the observation of red-shifted features. However, Fig. 1b indicates that the accretion emission only affects low-velocity features, so the implications for the lack of higher-velocity red-shifted components could be discussed more explicitly.

AN as Authors: We agree with the referee, and this is a question that was also posed by Referee #3. Our new section on the interpretation of the preferential blue-shift (see above) now includes these

considerations. As we have also written in our reply to Referee #3, we have been carrying out follow-up observations with the UVES spectrograph, and we now believe that we have two examples of clear red-shifted events (see Additional Figures attached to our comment to Referee #3), so the blue-shift may not be ubiquitous, even if they are more common.

Reviewer #5: Page 32:
Fig. 6:
It is unclear how the numbers in the figure are assigned; please clarify in the caption.

AN as Authors: The numbers in the figure were assigned from most blue-shifted (1) to least blue-shifted (highest number) in each epoch. However, this figure has been merged with current Fig. 1 and Extended Data Fig. 7, and the features are now assigned with letters and colors, from most blue-shifted (a) to least blue-shifted to make this more clear (see Fig. 2 and Extended Data Fig. 7).

Reviewer #5: Page 33:
Fig. 7b-d:
The paper would benefit from an equivalent figure with eccentricity vs semimajor axis for these simulations. Additionally, it could be helpful to overplot in grey the orbital parameters of the planetesimals that survive at the end of the 5Myr as suggested in one of my earlier comments.

AN as Authors: We refer the referee to our reply to comment “i) First, how many particles are scattered…” above, where we address this suggestion.

Reviewer #5: Typos:

Page 6:
Line 249:
“...as being a fraction the mass…” → “...as being a fraction of the mass…”

AN as Authors: This was modified in the text (see Section “Disc winds?”).

Responses to Comments from NEW Reviewer #6

Reviewer #6 (Remarks to the Author): I co-reviewed this manuscript with one of the reviewers who provided the listed reports. This is part of the Nature Communications initiative to facilitate training in peer review and to provide appropriate recognition for Early Career Researchers who co-review manuscripts.

AN as Authors: We sincerely thank the referee for their review of our manuscript, as well as the other referees.

Responses to Comments from Reviewer #3

Reviewer #3 (Remarks to the Author): The PDS 70 system is a young T Tauri star surrounded by a transition disc of gas and dust with ongoing planet formation as evidenced by the detection of two protoplanets. The system also shows the presence of water in the inner regions of the disc in JWST observations. In the present study the authors have found variable blue-shifted absorption lines of neutral sodium in HARPS spectra of the PDS 70 system. The author performed a detailed analysis of these lines and assessed several hyphotheses to explain the most likely origin of these sodium absorptions.The strongest contenders are disc winds and sublimating exocomets, and the authors conclude that exocomets on highly elliptical orbits would be the most likely scenario. This possibility could be connected to water delivery to the inner region of this system and therefore provide significant insight on the transport of water towards rocky planet during early stages of planet formation.

I see that the authors have fully taken into account the comments provided by the reviewers and have carefully addressed all concerns. The paper has been significantly improved, analysis and results are presented cautiously and meticulously, addressing sources of uncertainty, limitations and caveats, which overall paints a clearer picture on the plausibility of each scenario. I am satisfied with this version of the paper, the way it is now presented, and the answers to the referees comments. I now see the exocomet scenario as more plausible under the evidence of day-to-day strong variability being uncommon in disc winds. Furthermore, it is exciting to know that the authors have now obtained evidence of red-shifted absorptions, which further strengthen the exocomet scenario.

I only have a couple of minor comments. I would perhaps add a question mark at the end of the title since exocometary activity is not yet unambiguously confirmed.

AN as Authors: We agree with changing the title of the manuscript.

And, at the end of the "Stationary sodium lines" section, perhaps the authors could quickly assess the origin of the -22 and -16 km/s (pseudo) stationary lines. This could be easily done by checking the archival spectra of other stars at a small angular separation and similar distance from Earth (if possible); if any of these lines are observed in other star this provides a strong indication of the component originating from the ISM. See for instance the paper by Iglesias et al. (2023) on the blue-shifted lines in eta Tel. It would not take too much work and this could provide a clearer picture on the origin of these two absorptions.

AN as Authors: We found that a study by Crawford (1991) measured interstellar Na I lines towards several stars in the Scorpio-Centaurus association, where PDS 70 is located (Galactic Latitude of 19.20844 deg and Longitude of 318.06196 deg), including stars at small angular separations from PDS 70, the closest separation being μ Cen (3.6 deg) and φ Cen (2.0 deg). Their Fig. 2 and Table 2 show that ISM Na I lines are detected at heliocentric velocities around -10 km/s towards μ Cen and φ Cen. This value matches the velocity of one of our stationary Na I lines at -16 km/s in the stellar rest frame (which converts to a heliocentric velocity of roughly -10 km/s).

We have included this finding in the second paragraph of section “Stationary sodium lines”.

Given that the authors now have detected red-shifted sodium absorption in new UVES observations, I wonder if it wouldn't be preferable to hold the paper for a bit and re-submit once the UVES observations have been analysed so they could provide stronger evidence supporting the exocomet scenario. This is up to the authors of course, but I think this would be worth including in the paper as it strengthen the conclusions significantly.

<u>AN as Authors:</u> We agree that presenting part of our UVES dataset with red-shifted absorption components would significantly strengthen our work, and we have included part of our UVES spectra in the manuscript (see cover letter to editor/referees at the top of this document. Additionally see Fig. 6). However, we would prefer to analyse the full UVES dataset in depth in a future study, for many reasons:

– Firstly, because we want to give enough focus on the archival HARPS data of the current manuscript. We believe that including a detailed analysis of the UVES dataset in the current manuscript would shift the focus on the analysis away from the archival HARPS data. The 2018 archival HARPS data on its own has been the main driver to develop the exocomet scenario, and even though new observations strengthen the current study, the 2018 HARPS data should still be the primary focus of this manuscript.

– Secondly because, as it stands, the manuscript is already at the limit of word count and number of figures (see cover letter at the top of this file).

– Thirdly, because the UVES programme consists of over 50 observations which are still ongoing. A thorough analysis of this dataset will take a significant amount of time. Therefore, we would prefer to discuss these data in a follow-up manuscript, which is currently in preparation, providing the UVES data the appropriate focus.

We therefore decided to include part of the UVES spectra in this manuscript that we have already analysed, with the purpose of solely showing the presence of red-shifted Na I absorption lines, leaving an in-depth analysis of this dataset for a future manuscript.

Finally, congratulations on your work. I have no further comments, the paper has been thoroughly improved in my opinion.

<u>AN as Authors:</u> Finally, we sincerely thank the referee for acknowledging our modifications to the manuscript, for providing helpful feedback in the current and past versions, and for being satisfied with the current version.

Responses to Comments from Reviewer #4

Reviewer #4 (Remarks to the Author): The paper entitled "Sublimating exocomets in the PDS 70 system" by Novais et al. presents the spectroscopic detection of exocomets transiting the young protostar PDS 70.

The results presented in this manuscript are of major importance, as they put forward a plausible and physically consistent scenario for the origin of the water detected by JWST in this well-studied young planetary system. More broadly, given that only a small number of exocometary systems have been identified so far, PDS 70 represents a particularly valuable laboratory for the study of exocomets. With its high rate of exocomet transits, further supported by the authors' most recent UVES observations, this system is likely to become a prime target for future observations.

In the previous round of review, several important concerns were raised, in particular the possibility that the detected spectroscopic absorptions could instead originate from stellar winds. The authors have addressed these issues convincingly, notably through new UVES observational data showing redshifted absorption features, and a more cautious wording of the interpretative statements. These revisions substantially strengthen the manuscript and make the proposed exocometary interpretation more compelling.

In my view, the most important new element is the recent UVES observations showing redshifted absorptions, which provide additional support for the exocomet scenario. However, this key information is currently presented only in the response to the referees and is not made available to the readers of the paper. I therefore strongly encourage the authors to include this material, or at least a clear summary of it, in the manuscript.

Overall, the authors have satisfactorily addressed the comments raised on the previous version, and I have no major remaining concerns. In particular, the description of the fits to the absorption lines has been significantly improved, especially with Figures 2 and 7, and the new Tables 3 and 4 are welcome additions. I also appreciate the estimate of the total sodium mass converted into the typical size of an evaporated planetesimal, of about 1 km in diameter, which is consistent with the exocomet scenario.

The revised Figures 2 and 7 are useful additions, as they allow the reader to identify more clearly each detected feature and its measured properties. Tables 3 and 4 are also valuable, although they remain somewhat difficult to read in their current format. I suggest making them available in electronic form.

In short, I recommend publication of the manuscript after minor revisions.

In particular, I suggest

1) To include in the manuscript a description, or at least a clear summary, of the most recent UVES observations showing redshifted absorption features, since these provide important additional support for the exocomet scenario.

AN as Authors: We agree that the 2026 UVES data provide important support the exocomet scenario and we therefore agree to include part of the 2026 UVES observations in the manuscript, e.g. displaying the data in an additional figure (see cover letter to editor/referees at the top of this document, and additionally see Fig. 6), while stating that this dataset will be examined in detail in a future study (see reply to reviewer #3). At the time of writing, the observations are not yet completed and a coherent analysis will be carried out over the course of the coming year.

2) To make Tables 3 and 4 available in electronic form, as they contain useful information but are difficult to read in their current format.

AN as Authors: We are now providing Tables 2, 3, and 4 in a csv format (see supplementary files, also available at https://codeocean.com/capsule/4125005/tree) as well as presenting them in the manuscript.

3) To ensure that the wording throughout the manuscript remains appropriately cautious when discussing the exocomet interpretation.

AN as Authors: We have proof-read our manuscript to make sure that our text is appropriately cautious when addressing exocomets as a plausible interpretation while still discussing alternative scenarios (e.g. disc winds) and clearly stating all necessary measurements to unambiguously confirm exocomet activity in PDS 70. We have additionally modified the title of the manuscript, as suggested by Reviewer #3, to further moderate our conclusions.

Finally, we sincerely thank the referee for their helpful insights during the revision process, and for recommending our manuscript for publication.

Reviewer #4 (Remarks on code availability): In contrast to the previous review round, I was unable to access the sodium-line fitting code used in this work, despite the authors' statement that it is publicly available through Code Ocean at https://codeocean.com.

AN as Authors: Due to a quota limit to run our code in the free version of Code Ocean, earlier we had to make a new Code Ocean capsule to provide our fitting code. We ask the referee to please try accessing the code again using the following url: https://codeocean.com/capsule/4125005/tree.

Responses to Comments from Reviewer #5

Reviewer #5 (Remarks to the Author): Dear authors,
Thank you for your careful revision and for thoroughly addressing the concerns raised in the previous referee report. The authors have responded to all points raised, and the improvements are clearly reflected in the manuscript. The clarified plots, the more detailed explanation of the observed blueshifted lines, and the restructuring and reordering of the sections have all contributed to making this a significantly stronger manuscript.

A few minor clarifications would further strengthen this work. The remaining points to clarify are outlined below.

I co-reviewed this manuscript with one of the reviewers who provided the listed reports. This is part of the Nature Communications initiative to facilitate training in peer review and to provide appropriate recognition for Early Career Researchers who co-review manuscripts.

Minor points linked to the authors' response to the referee reports:

Page 9
Section: "Exocomet as the origin of the variable sodium lines"

Paragraph 1: The minimum sodium mass estimates quoted here would benefit from a clearer justification of how these values are derived. A reference to the relevant section in the manuscript would help.

AN as Authors: The sodium mass estimates cited in this paragraph are derived by dividing the minimum sodium cloud masses of 1.3e8–3.9e9 kg (derived in "Calculation of sodium cloud mass" and quoted in the manuscript) by the abundance of sodium in CI Chondrite meteorites of 5.01e-3 (Lodders 2003). We included a new Method section "Estimation of planetesimal size" explicitly showing this calculation, and referenced the section at the end of this paragraph. Note that, in this new section, we also included an estimate assuming a comet bulk density, which yields similar planetesimal diameters (between 0.5–1.4 km).

Paragraph 2: The discussion comparing PDS 70 and Beta Pic would benefit from some restructuring to improve clarity. As currently written, it is stated that Beta Pic ionises sodium more efficiently than PDS 70, yet sodium absorption features appear to be clearer and more frequent in PDS 70. A clearer explanation of why this is the case would help the reader.

We suggest reorganising this section as follows. When introducing Beta Pic, the discussion primarily focuses on the CaII H&K lines. It would therefore be natural to first address the lack of detected variability in these lines for PDS 70. The discussion can then transition to the sodium absorption features detected around Beta Pic and how they compare to those reported in this work (e.g., differences in detection frequency and possible explanations for them). Together, these points can then be presented as a source of uncertainty regarding the exocometary origin of the signal.

AN as Authors: We explain the difference between Beta Pictoris and PDS 70 as follows: In PDS 70 we observe neutral sodium, which is mainly absent in Beta Pictoris exocomets due to the fact there, sodium is much easier to ionise due to the fact that Beta Pic is a hot, A-type star, and exocomets inferred to be located very close to the star (e.g. Beust et al. 2024). Efficient ionisation in Beta Pictoris therefore makes the detection of neutral sodium (Na I) more rare. In PDS 70 (cooler star), sodium is less ionised, and can therefore be more easily detected in its neutral state.

We have clarified and reorganised the paragraph as requested, presenting the Ca II exocomet detections in Beta Pic, then addressing the fact that we do not see exocomets in Ca II in PDS 70, and finally addressing the Na I detections and the difference in ionisation efficiency (see paragraph "We compare our findings with…" in section "Exocomets as the origin of the variable sodium lines").

Page 29
Section: "N-body simulations"

The multiple changes to the text and answer to the previous comments help to clarify most of the issues that were raised before. However, a few minor points should still be addressed:

- Text should say that stopping timescales are longer than the age of the system for planetesimals larger than "x" km. The absorption features could be caused by planetesimals/exocomets with a wide range of sizes, and so it is important to say for what planetesimal sizes the N-body simulations are valid.

<u>AN as Authors:</u> We have now included the size of the planetesimals used in the calculation (0.3 km or larger) in the text (see section "N-body simulations", second paragraph "The goal of our simulation…").

- The new panels in Figure 4 further clarify the dynamical picture of how particles are evolving and transported to the inner system. However, there are still details that need to be clarified. Mainly, how are particles far from the chaotic zone of c excited into highly eccentric orbits before experiencing close encounters / scattering events with planet c? Panel c in Figure 4 shows that there are regions in the outer disk where particles can be excited, while this does not happen in other regions. For example, there is a "gap" between 70 and 80 au where no particles are scattered to the inner regions. With 3 planets, this changes, and no particles further than 75 are scattered inwards. Mean motion or secular resonances could be responsible for exciting those particles far from the inner edge of the outer disk, which could explain why there are gaps or islands in panels c and f. The authors could plot the eccentricity vs semi-major axis as a function of time in the outer disk, overplot the MMRs of c and even the secular resonances for the 2 planet case. Alternatively, the text could highlight these gaps/islands and list what processes may be exciting those particles further out. Note that the excitation will compete with gas drag, and so this process might preferentially excite only the largest planetesimals.

<u>AN as Authors:</u> We agree with the referee that these details about the dynamics of N-body simulation were not fully explored in our manuscript. Unfortunately, due to the large number of particles, it was unfeasible to save particle parameters as a function of time, which would generate a very large volume of data. Therefore, we are not able to plot the eccentricity vs. semi-major axis of all particles as a function of time.

Instead, as suggested by the referee, we overplotted the strongest mean-motion resonances with PDS 70 c in panels c and g of Fig. 4. We additionally added panels d and h, showing the fate of particles that initialised at each semi-major axis between 54–87 AU (outer disc), also with the MMRs with PDS 70 c overplotted. The new panels d and h clearly show that spikes in scatterings (inward and outward) coincide with several low-order resonances, suggesting that MMR with planet c are indeed a likely driver for the scattering of planetesimals towards the inner disc. We included this discussion in section "N-body simulations", see last paragraph "In addition to the findings presented…".

As for the role of gas drag, a computation of the stopping time in the mid-plane of an alpha-disc model indicates that the stopping time is orders of magnitude larger than the age of the system for planetesimals of 0.3 km and larger in the outer disc beyond 50 AU. We therefore believe that – at the very least in the outer disc, it is safe to neglect the effects of gas drag.

- The choice of recording particle parameters at the time they cross the gap, rather than at the end of the simulation, is acceptable provided appropriate caveats are given. The uncertainty introduced by the N-body simulations is acknowledged in lines 574-585, which is good. However, the “N-body simulation” section would benefit from a more explicit justification for this choice – specifically, clarifying that subsequent orbital evolution after “crossing the gap” is not the focus, and that the primary goal is simply to demonstrate that material transport from the outer to inner disc is possible.

AN as Authors: We fully agree that the justification for this choice is not explicit in the most recent version of the manuscript. This was present in previous versions of the manuscript and ended up being commented out of the current version. We have now complemented the second paragraph of section “N-body simulations” with this justification, using text from the previous version of the manuscript.

- The choice of initial particle inclinations is well investigated and clearly justified in the authors’ rebuttal. It would nonetheless be worthwhile to include a brief justification in the manuscript itself for the adopted range of ±10deg. This could be as simple as a short statement acknowledging that similar conclusions are reached with lower initial inclinations.

AN as Authors: We have now justified the choice of initial particle inclinations in the section “N-body simulations” (see third paragraph “We simulate a total of 10 000 massless test particles…”), motivating the choice with the inclinations in the present-day Kuiper belt (Gulbis et al. 2010). We also stated that we also tested lower inclinations closer to the mid-plane, which yielded similar results, likely because the planets are not perfectly coplanar (Trevascus et al. 2025), and even a small mutual inclination between the planets is sufficient to excite particles vertically.

Responses to Comments from Reviewer #6

Reviewer #6 (Remarks to the Author): I co-reviewed this manuscript with one of the reviewers who provided the listed reports. This is part of the Nature Communications initiative to facilitate training in peer review and to provide appropriate recognition for Early Career Researchers who co-review manuscripts.

AN as Authors: We thank the referee for their review of our manuscript, as well as the other referees.

NCOMMS-25-98380-C
"Potential sublimating exocomets around the young star PDS 70"

Responses to Comments from Reviewer #3

Reviewer #3 (Remarks to the Author): Dear authors and editors,

I am happy with the current version of the manuscript and recommend for publication.
I have no further comments. Congratulations on your work. Looking forward to see what the analysis of the full UVES dataset reveals.

Kind regards

AN as Authors: We sincerely thank the referee for their previous insights, which helped improve our manuscript, and for recommending our manuscript for publication.

Responses to Comments from Reviewer #4

Reviewer #4 (Remarks to the Author): The paper entitled "Sublimating exocomets in the PDS 70 system" by Novais et al. presents the spectroscopic detection of exocomets transiting the young protostar PDS 70.

The authors have now addressed all the major and minor comments raised by the reviewers. The manuscript presents a detailed and convincing case in support of the exocomet scenario. In particular, the inclusion of UVES spectra in the new Figure 6, as suggested by Reviewers 3 and 4, is a valuable addition.

I recommend publication of the manuscript in Nature without further revisions.

AN as Authors: We sincerely thank the referee for providing helpful feedback and for recommending our manuscript for publication.

Reviewer #4 (Remarks on code availability): With the new link provided by the authors, I am now able to access the codes. I verified that the code is publicly available and that all required files appear to be included. However, I did not attempt to run it or to assess its proper functioning.

AN as Authors: We thank the referee for verifying our code's accessibility.

Responses to Comments from Reviewer #5

Reviewer #5 (Remarks to the Author): Dear authors,
Thank you for your careful revision and for thoroughly addressing the concerns raised in the previous referee report, particularly those regarding more explicit caveats. The paper has improved significantly and now satisfies all our previous concerns.

We noticed one remaining typo in a newly added section (page 30, line 1358: missing "[in] the inner regions…"). Once this is corrected, the paper will be ready for publication.

Congratulations on a very nice piece of work, which will generate great excitement in the community.

I co-reviewed this manuscript with one of the reviewers who provided the listed reports. This is part of the Nature Communications initiative to facilitate training in peer review and to provide appropriate recognition for Early Career Researchers who co-review manuscripts.

AN as Authors: We sincerely thank the referee for providing improvements to the manuscript, and for acknowledging our work on this study. We also thank the reviewer for spotting a typo in page 30, line 1358, which is now fixed.

Responses to Comments from Reviewer #6

Reviewer #6 (Remarks to the Author): I co-reviewed this manuscript with one of the reviewers who provided the listed reports. This is part of the Nature Communications initiative to facilitate training in peer review and to provide appropriate recognition for Early Career Researchers who co-review manuscripts.

AN as Authors: We sincerely thank the referee for their review of our manuscript, as well as all the other referees.
